# Mechanisms of telomerase inhibition by oxidized and therapeutic dNTPs

**DOI:** 10.1038/s41467-020-19115-y

**Published:** 2020-10-20

**Authors:** Samantha L. Sanford, Griffin A. Welfer, Bret D. Freudenthal, Patricia L. Opresko

**Affiliations:** 1 Department of Environmental and Occupational Health, University of Pittsburgh Graduate School of Public Health and UPMC Hillman Cancer Center, Pittsburgh, PA USA; 2 Department of Biochemistry and Molecular Biology, University of Kansas Medical Center, Kansas City, KS USA; 3 Center for Nucleic Acids Science and Technology, Carnegie Mellon University, Pittsburgh, PA USA

**Keywords:** Enzyme mechanisms, Chemotherapy, Telomeres, DNA synthesis

## Abstract

Telomerase is a specialized reverse transcriptase that adds GGTTAG repeats to chromosome ends and is upregulated in most human cancers to enable limitless proliferation. Here, we uncover two distinct mechanisms by which naturally occurring oxidized dNTPs and therapeutic dNTPs inhibit telomerase-mediated telomere elongation. We conduct a series of direct telomerase extension assays in the presence of modified dNTPs on various telomeric substrates. We provide direct evidence that telomerase can add the nucleotide reverse transcriptase inhibitors ddITP and AZT-TP to the telomeric end, causing chain termination. In contrast, telomerase continues elongation after inserting oxidized 2-OH-dATP or therapeutic 6-thio-dGTP, but insertion disrupts translocation and inhibits further repeat addition. Kinetics reveal that telomerase poorly selects against 6-thio-dGTP, inserting with similar catalytic efficiency as dGTP. Furthermore, telomerase processivity factor POT1-TPP1 fails to restore processive elongation in the presence of inhibitory dNTPs. These findings reveal mechanisms for targeting telomerase with modified dNTPs in cancer therapy.

## Abstract (web summary)

Telomerase enzymes add telomeric repeats to the end of linear chromosomes. Here the authors reveal mechanisms by which oxidized dNTPs and therapeutic dNTPs inhibit telomerase-mediated telomere elongation.

## Introduction

Telomeres cap linear chromosome ends, and in humans it consist of GGTTAG repeats and a protective protein complex called shelterin^1^. Critically short telomeres trigger a DNA damage response and cell senescence which contribute to aging-related diseases^2^. Telomeres shorten during each replication due to the end replication problem^3^. The ribonucleoprotein complex, telomerase, counteracts this problem by adding telomeric repeats to chromosome ends^4^. Human telomerase consists of two main components, a reverse transcriptase (TERT) and a functional RNA (TR) which contains the template sequence for synthesis of GGTTAG repeats^5^. Telomerase is expressed in human germ and stem cells, and is upregulated in over 85% of cancers, enabling unlimited proliferation and tumorigenesis^6,7^. Therefore, inhibiting telomerase activity is a promising therapeutic strategy to treat many cancers.

**Fig. 1:**
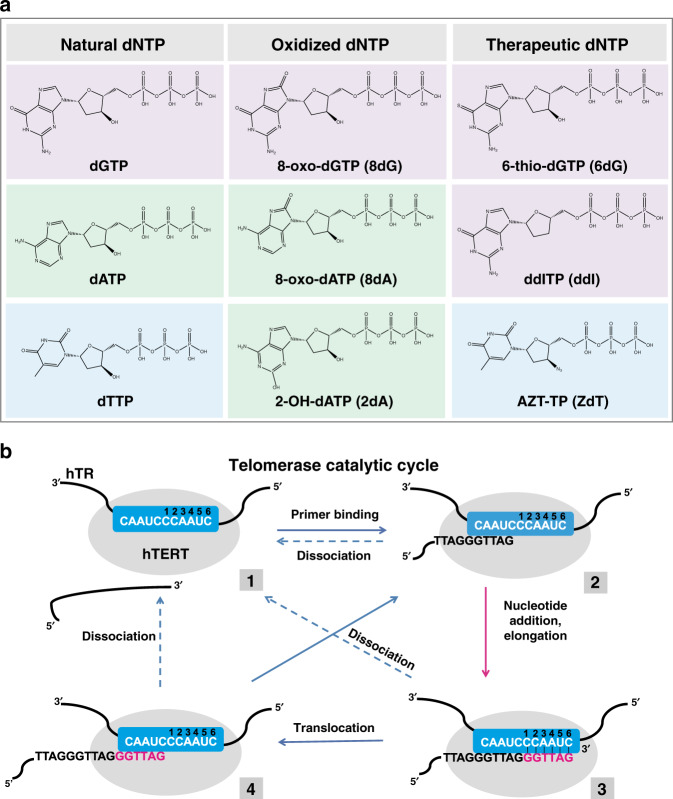
dNTPs used in telomerase reactions. **a** Chemical structures of dNTP analogs used in this study and the corresponding natural dNTP shown in the same color. **b** Telomerase catalytic cycle. Blue indicates the telomerase RNA template; black indicates DNA primer; red indicates newly added nucleotides; numbers represent the steps in the cycle.

Numerous studies show that oxidative stress causes accelerated telomere shortening^8^. Oxidative stress is an imbalance between reactive oxygen species (ROS) and antioxidant defense systems in the cell. ROS are highly reactive toward biomolecules and cellular components including lipids, proteins, and nucleic acids. Free deoxynucleotide triphosphates (dNTPs) are highly susceptible to oxidative damage from ROS, and insertion of oxidized dNTPs into the genome by DNA polymerases during replication or repair is mutagenic and toxic to cells^9,10^. To prevent this, MutT homolog 1 (MTH1) hydrolyzes oxidized dNTPs (e.g. 8-oxo-dGTP, 2-OH-dATP, and 8-oxo-dATP) (Fig. 1a), and is a target for cancer therapy since cancer cells are thought to have higher levels of oxidized dNTPs and are more sensitive to MTH1 inhibition^10,11^. MTH1 depletion inhibits telomere maintenance and telomerase activity in cancer cells under oxidative stress conditions, and telomerase insertion of 8-oxo-dGTP terminates further telomere elongation in vitro^12–14^. However, MTH1 also removes oxidized dATPs, but whether these damaged nucleotides can inhibit telomerase, similar to 8-oxo-dGTP, and contribute to telomere shortening is unknown.

While oxidized dNTPs arise naturally, synthetic modified dNTPs and nucleoside analogs have a long history of successful use for anti-viral and cancer therapies. Nucleoside reverse transcriptase inhibitors (NRTIs) block human immunodeficiency virus (HIV) reverse transcriptase (RT) to prevent viral replication. In their phosphorylated form, NRTIs compete with natural dNTPs for insertion by HIV-1 RT, and act as chain terminators^15^. The catalytic core of TERT is structurally homologous to the HIV-1 RT domain^16^, and telomerase inhibition has been proposed to contribute to premature aging observed in HIV patients undergoing long-term NRTI therapy^17,18^. While studies confirmed that various NRTIs cause telomere shortening and inhibit telomerase in vitro^19–21^, whether telomerase can insert these chain-terminating analogs during telomere synthesis was unknown. Thiopurines are another class of therapeutic nucleoside analogs that cause telomere shortening, and are currently used in clinical practice as antileukemic, anti-inflammatory, and immunosuppressive agents^22–24^. 6-thio-dGTP, which forms in cells from 6-thio-dG, promotes cell death in telomerase-positive cancer cells but not in normal cells, and decreases tumor growth in mouse xenograft studies^25^. While 6-thio-dG causes telomere shortening and dysfunction, the mechanism is unclear because extracts from treated cells do not show reduced telomerase activity^25^. Furthermore, since both NRTIs and thiopurines can elevate ROS by causing mitochondrial dysfunction, they may increase oxidative damage within nucleotide pools^26,27^.

Here, we systematically investigate how oxidized dATPs (2-OH-dATP and 8-oxo-dATP) and synthetic therapeutic dNTPs (derived from 6-thio-dG, didanosine (ddITP), and azidothymidine (AZT-TP)) (Fig. 1a) impact telomerase activity, to determine how these modified dNTPs drive telomere shortening and dysfunction. We conduct a series of direct telomerase extension assays in the presence of modified dNTPs on various telomeric substrates to define mechanisms of inhibition. We find that oxidized dATPs also inhibit telomerase activity, but unlike 8-oxo-dGTP, 2-OH-dATP is not mutagenic or a chain terminator. Rather, our data indicate 2-OH-dATP impedes telomerase translocation and further nucleotide and repeat addition. 6-thio-dGTP strongly inhibits telomerase by disrupting translocation, similar to 2-OH-dATP. Finally, we demonstrate that telomerase inserts NRTIs ddITP and AZT-TP during DNA synthesis, and that these analogs are genuine chain terminators. Cumulatively, we uncover two distinct mechanisms by which oxidized and therapeutic dNTPs promote telomere shortening.

## Results

### Modified dNTPs decrease telomerase processivity

To determine if telomerase can utilize oxidatively damaged 2-OH-dATP and 8-oxo-dATP or therapeutic NRTIs and 6-thio-dGTP (Fig. 1a) for telomere elongation, we conducted direct repeat addition assays. For NRTIs, we selected ddITP and AZT-TP based on evidence they promote telomere shortening^20,21,28^. The telomerase catalytic cycle starts when the telomeric single strand overhang base pairs with the complementary 3′ end of the telomerase RNA template priming synthesis^5^. Upon incorporation of the incoming dNTP, the telomerase active site moves to the next template base and telomere elongation continues until the template 5′ boundary is reached for processive nucleotide addition (Fig. 1b, steps 2 and 3). In human telomerase, the template region is 11 nt (3′-rCAAUCCCAAUC-5′) comprising of an alignment region plus a template sequence (numbered 1–6 in Fig. 1b). Then, telomerase can either dissociate or translocate on the DNA product, generating a realigned 5-bp RNA:DNA hybrid for processive repeat addition. The 6-nt RNA template is reverse transcribed for each cycle of repeat addition and template recycling (Fig. 1b, steps 2–4). Nucleotide addition processivity (NAP) is the number of nucleotides added prior to enzyme dissociation from the template, and repeat addition processivity (RAP) is the number of repeats added prior to dissociation.

**Fig. 2:**
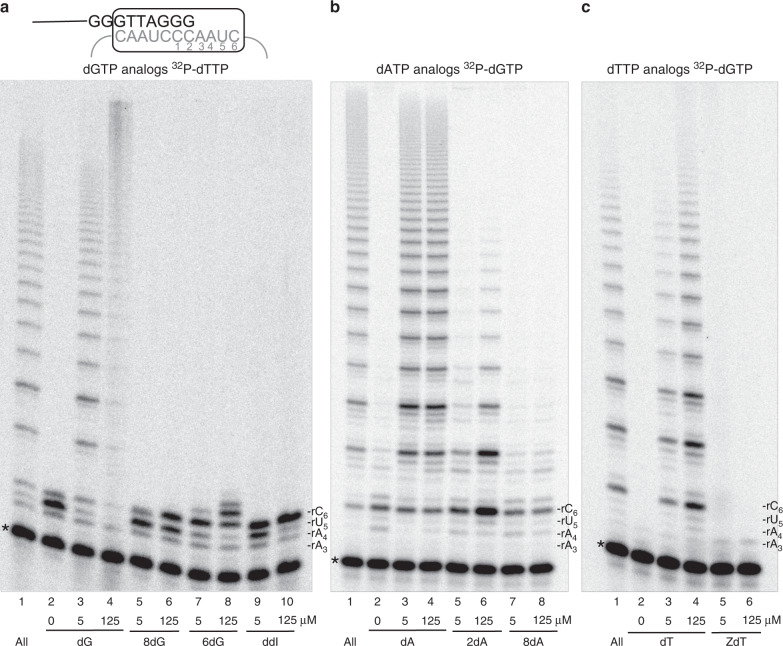
Oxidized and therapeutic dNTPs inhibit telomerase processivity. Telomerase reactions were conducted with (TTAGGG)_3_ primer and cellular-concentration dNTPs except that the indicated natural or modified dNTP was titrated in at increasing concentrations (0, 5, 125 μM) along with either 0.3 μM [α-^32^P]dTTP **a** or 0.3 μM [α-^32^P]dGTP **b**, **c** to label the products. The loading control (*) was a ^32^P-end labeled 18-mer oligonucleotide. Numbers on the left indicate the number of added repeats; letters on the right indicate template residue. Images are representative of three independent experiments.

For telomerase reactions, we used the standard substrate of three TTAGGG repeats and immunopurified FLAG-tagged telomerase overexpressed in human HEK 293T cells. This preparation, termed super telomerase, has kinetic properties similar to endogenous telomerase (Supplementary Fig. 1)^29–31^. We conducted the reactions with cellular relevant dNTP concentrations (24 μM dATP, 29 μM dCTP, 37 μM dTTP, 5.2 μM dGTP, averaged from multiple studies^32^), since dNTP pool balance can impact DNA synthesis^32^. We replaced increasing concentrations of the natural dNTP with the corresponding oxidized or therapeutic dNTP analog (0.5–125 μM) in reactions containing the remaining three natural dNTPs. Repeat processivity was measured using the convention of calculating the number of repeats synthesized before half of the DNA substrates dissociate from telomerase^33^ (Supplementary Fig. 2). 8-oxo-dGTP served as a control since it is an established telomerase chain terminator^12^. Increasing dGTP, dATP, or dTTP greatly increased repeat processivity as evidenced by the appearance of longer products (Fig. 2). Loss of signal at the highest dGTP concentration was due to dGTP outcompeting the low radioactive dTTP amounts used to label products, and was not observed in dATP or dTTP titration reactions containing radio-labeled dGTP (Fig. 2 and Supplementary Fig. 2). Similar to 8-oxo-dGTP, 8-oxo-dATP failed to support processive repeat addition (Fig. 2a, b). In contrast, we observed moderate telomeric synthesis in the presence of 2-OH-dATP, but repeat processivity was lower than reactions with unmodified dATP (Fig. 2b and Supplementary Fig. 2). These data indicate that oxidatively damaged dATPs inhibit telomerase, although to a different extent depending on the modification.

Next, we examined whether telomerase could utilize therapeutic dNTPs for elongation. Reactions with 6-thio-dGTP showed only one repeat added, although at the highest 6-thio-dGTP concentrations, telomerase incorporated two additional nucleotides after translocation at the rC_1_rC_2_ positions (Fig. 2a, lanes 7 and 8). This suggests 6-thio-dGTP inhibits both nucleotide and repeat processivity. Although the NRTI didanosine is an adenine analog, it is converted to either ddATP or ddITP, which is an isoguanine^34,35^. When ddITP replaced dGTP in the reaction, we observed no synthesis past the first rC_6_ template even with increasing ddITP concentrations (Fig. 2a, lanes 9 and 10). Virtually no products were observed in reactions containing AZT-TP, although incorporation opposite the first rA_3_ template blocks further extension (Fig. 2c). In summary, the aborted extension products show substitution of the natural dNTPs with 8-oxo-dNTPs inhibit telomerase to a similar extent as substitution with HIV-RT chain terminators or 6-thio-dGTP. Our data suggest that either telomerase cannot incorporate these modified dNTPs during telomeric DNA synthesis, or following incorporation, they inhibit further elongation of the telomere.

### Telomerase inhibition mechanism depends on the modified dNTP

**Fig. 3:**
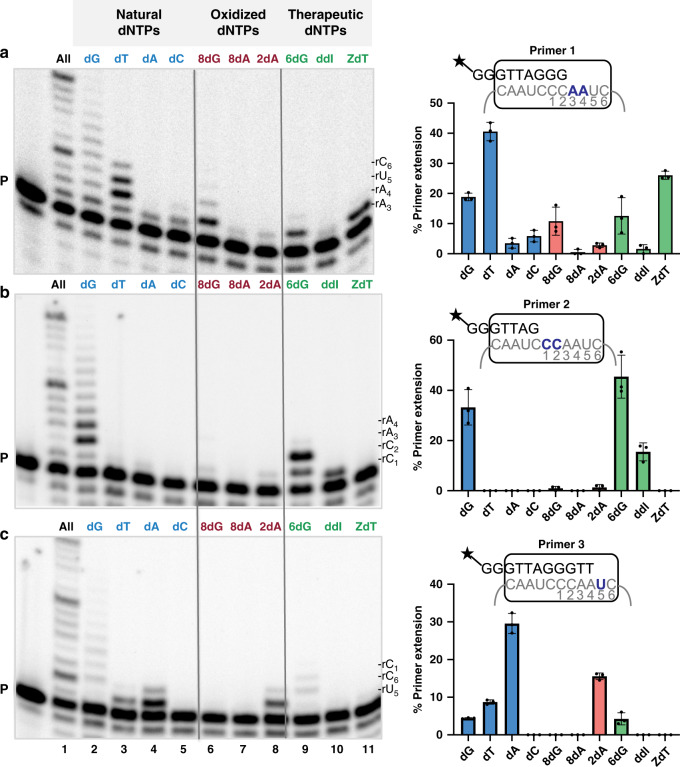
Telomerase insertion of modified dNTPs. Telomerase reactions were conducted with 5 nM ^32^P-end labeled primer **a** Primer 1 (TTAGGG)_3_, **b** Primer 2 (GGTTAG)_3_, or **c** Primer 3 (AGGGTT)_3_. Reactions contained cellular-concentration dNTPs (lane 1) or 50 μM of indicated natural dNTP (blue, lanes 2–5), oxidized dNTP (red, lanes 6–8), or therapeutic dNTP (green, lanes 9–11). Products were separated on denaturing gels. 8dG (8-oxo-dGTP); 8dA (8-oxo-dATP); 2dA (2-OH-dATP); 6dG (6-thio-dGTP); ddI (ddITP); ZdT (AZT-TP). Letters on the right indicate the template base; P indicates unextended 18-mer primer. Graphs represent the percent of total primers extended. Images are representative of, and data are shown as mean ± s.d. from, three independent experiments. Source data are provided as a Source Data file.

A chain terminating mechanism of inhibition requires that the enzyme adds the modified dNTP to a growing chain, which then blocks further synthesis. Since we showed previously that 8-oxo-dGTP is a telomerase chain terminator, we used it as a positive control^12^. To test whether telomerase can incorporate other modified dNTPs, and whether the incorporation is chain terminating and/or mutagenic, we conducted direct extension assays in which we radiolabeled the primer and added increasing concentrations (5, 50, and 500 μM) of a single dNTP (Supplementary Figs. 3, 5, 6, and 7). Since modified dNTPs may prefer mispairing, we tested various primers in which the first template base was either rA_3_, rC_1_, or rU_5_ (primer 1, primer 2, and primer 3, respectively), and compared the percent primer extension for each dNTP at the middle 50 μM concentration (Fig. 3). Primers 1 and 2 initiate synthesis at consecutive rA_3_rA_4_ or rC_1_rC_2_ template bases, respectively, so we could examine both incorporation and extension to the next base. However, the telomerase template lacks tandem rU residues.

We first tested incorporation of natural dNTPs at the various template positions as controls. Reactions with a single dNTP type is an established method for determining DNA synthesis fidelity, defined as selectivity for incorporating a correct dNTP versus an incorrect or modified dNTP^36,37^. Interestingly, telomerase extended all three primers in the presence of dGTP, suggesting telomerase may incorporate dGTP opposite each template base even at low cellular concentrations (5 μM) (Supplementary Fig. 3). However, comparisons at 50 μM dGTP indicates a preference for correct incorporation over misincorporation in order of rC_1_ (33%, primer 2) > rA_3_ (19%, primer 1) > rU_5_ (4%, primer 3) extension (Fig. 3 lanes 2). We also observed telomerase synthesis of poly-dG ladders, as reported previously^12^, even at early time points and at a single rC (Supplementary Fig. 4a, b). As a control, reactions with DNA polymerase β showed no evidence of dGTP laddering or misincorporation opposite template A (Supplementary Fig. 4c, d). Poly-nucleotide ladders were not observed in reactions with any other dNTP (Fig. 3) or with RNase addition (Supplementary Fig. 3d). In the presence of dTTP, telomerase preferentially extended primer 1 showing incorporation opposite rA_3_ and strong termination after rA_4_, as expected, but also minor extension to the subsequent rU_5_ (Fig. 3a lane 3, and Supplementary Fig. 5). Consistent with this, telomerase could extend primer 3 by misincorporating dTTP opposite rU_5_ (Fig. 3c lane 3). Thus, while telomerase can misinsert dTTP, it strongly prefers correct insertion (41% primer 1 extension versus 9% primer 3 extension). Similarly, telomerase strongly preferred correct insertion of dATP opposite rU_5_, elongating primer 3 with minimal extension to the next rC_1_, and poor or no extension of primers 1 and 2, respectively (Fig. 3 lane 4). Finally, telomerase only extended primer 1 in the presence of dCTP, indicating some misincorporation opposite rA_3_ (Fig. 3 lane 5, Supplementary Fig. 5). In summary, telomerase extension was most accurate with primer 2 (template rC_1_) and least accurate with primer 1 (template rA_3_). Additionally, telomerase incorporation of dGTP is more error prone than the other natural dNTPs, as indicated by the production of poly d(G) ladders.

We next tested whether telomerase can incorporate oxidized dATPs onto the growing telomere chain using the end-labeled primers. Telomerase elongated primer 1 by misinserting 8-oxo-dGTP opposite rA_3_ with minimal extension to the next template rA_4_, confirming its chain-terminating ability (Fig. 3 lane 6 and Supplementary Fig. 6)^12^. Telomerase showed little to no extension of primers 2 and 3 with 8-oxo-dGTP, indicating poor incorporation opposite rC_1_ or rU_5_. In contrast, we detected virtually no extension of any primers with 8-oxo-dATP, except for minimal misinsertion opposite rA_3_ with primer 1 (Fig. 3 lane 7 and Supplementary Fig. 6), suggesting 8-oxo-dATP is a very poor substrate for telomerase. However, telomerase readily extended primer 3 by inserting 2-OH-dATP opposite the correct rU_5_, generating only 2-fold less product than with dATP (16% versus 30%, respectively), and showed minimal extension to the next incorrect rC_1_ position (Fig. 3 lane 8). Unlike 8-oxo-dGTP, telomerase insertion of 2-OH-dATP is not mutagenic, showing very low extension of primers 1 and 2 indicative of poor misinsertion opposite rA_3_ and rC_1_, respectively. The single template rU did not allow us to determine whether 2-OH-dATP insertion is chain terminating. In summary, our data indicate telomerase insertion of oxidized dNTPs follows the order 2-OH-dATP > 8-oxo-dGTP > 8-oxo-dATP opposite their preferred template base.

Analysis of the therapeutic dNTPs demonstrates that telomerase can add each of these analogs to a telomere chain. Regarding the NRTIs, telomerase inserted ddITP opposite rC_1_, extending primer 2 with moderate efficiency (16% extension) compared to reactions with dGTP (33% extension), but did not extend to the next rC_2_ (Fig. 3 lane 10 and Supplementary Fig. 7). Telomerase extended primer 1 with AZT-TP, showing correct insertion opposite rA_3_ with no extension to the next rA_4_; yielding 26% extension compared to 41% extension with dTTP (Fig. 3 lane 11). These data definitively show that these HIV-1 RT chain terminators are also genuine telomerase chain terminators, and define the inhibition mechanism. Strikingly, telomerase was able to extend all three primers with 6-thio-dGTP, showing a preference for incorporation in order of template rC_1_ (45%) > rA_3_ (13%) > rU_6_ (4%) primer extension (Fig. 3 lane 9), at product yields comparable to dGTP incorporation. In reactions with primer 2, telomerase correctly incorporated 6-thio-dGTP opposite rC_1_, showing strong termination after insertion opposite the next rC_2_. This suggests that, like dGTP, 6-thio-dGTP can bind at the telomerase active site at all template primer positions, however unlike dGTP, it does not support poly-nucleotide laddering. Our data indicate telomerase insertion of therapeutic dNTPs follows the order 6-thio-dGTP > AZT-TP > ddITP. Unlike the NRTIs and 8-oxo-dGTP, 6-thio-dGTP is not a telomerase chain terminator, indicating the mechanism of inhibition is distinct.

### 2-OH-dATP and 6-thio-dGTP insertion disrupt translocation

**Fig. 4:**
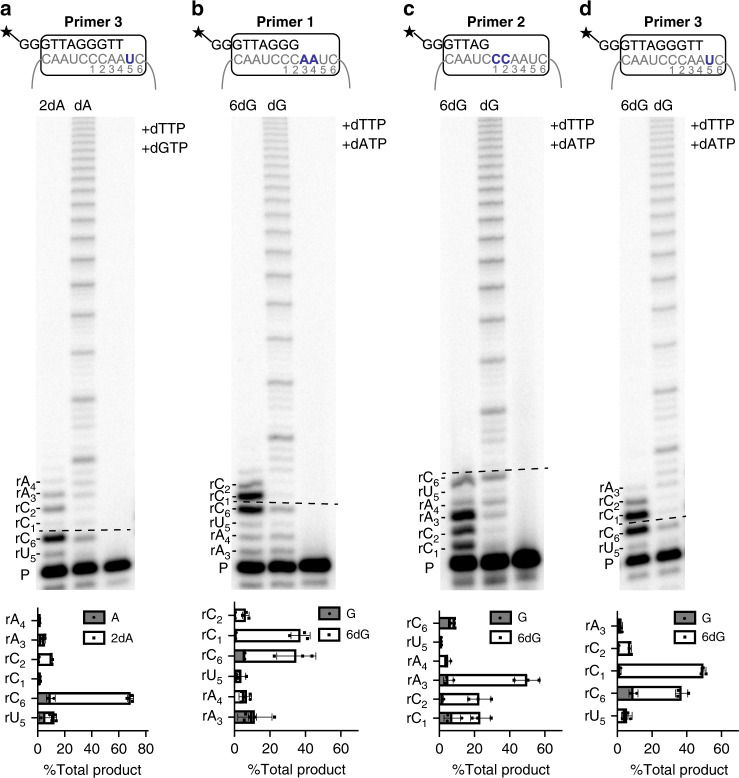
2-OH-dATP and 6-thio-dGTP insertion disrupt telomerase translocation. Direct telomerase assays were conducted with 5 nM ^32^P-end labeled primer **a** and **d** Primer 3 (AGGGTT)_3_, **b** Primer 1 (TTAGGG)_3_, or **c** Primer 2 (AGGGTT)_3_. Reactions contained 50 μM dATP, dTTP, and dGTP, except when 2-OH-dATP (2dA) or 6-thio-dGTP (6dG) was substituted for dATP or dGTP, respectively, where indicated. Products were separated on denaturing gels. Letters on the y-axis of the graph indicate the template base; P indicates unextended 18-mer primer. Graphs represent percent of product terminated at each template position as a function of total products. Images are representative of, and data are the mean ± s.d. from, three independent experiments. Source data are provided as a Source Date file.

To better understand how 2-OH-dATP and 6-thio-dGTP inhibit the telomerase catalytic cycle, we examined whether telomerase could incorporate a natural dNTP after adding the modified dNTP. First, we used primer 3 to examine extension after insertion opposite rU_5_ in reactions with 50 μM each of dTTP, dGTP, and either dATP or 2-OH-dATP. We observed typical processive synthesis with all three natural dNTPs, but replacing dATP with 2-OH-dATP generated a strong termination product after extension to the next base (rC_6_), which is the final base prior to translocation (Fig. 4a). These data indicate that telomerase can extend after insertion of 2-OH-dATP, but translocation to add the next repeat is greatly compromised. Those few reactions that continued after translocation terminated prior to reaching the template end, suggesting 2-OH-dATP disrupts both repeat and nucleotide processivity. Next, we conducted reactions with 50 μM each of dATP, dTTP, and either dGTP or 6-thio-dGTP. Figure 4b–d show typical processive synthesis with the natural dNTP after initiating synthesis opposite rA_3_ (primer 1), rC_1_ (primer 2), and rU_5_ (primer 3). Next we replaced dGTP with 6-thio-dGTP. Primer 1 reactions show strong termination products after telomerase inserted 6-thio-dGTP opposite rC_6_ position (4 nucleotides added) (Fig. 4b), suggesting dissociation prior to translocation. We also observed strong termination products at the next rC_1_ (5 nucleotides added), indicating either successful translocation in some reactions followed by dissociation, or template slippage for an additional 6-thio-dGTP incorporation prior to translocation. This result was recapitulated with primer 3. Figure 4d shows that after telomerase inserted dATP opposite rU_5_, the majority of the reactions terminated after 6-thio-dGTP insertion opposite rC_6_ or the next rC_1_. Finally, reactions with primer 2 show that telomerase incorporates 6-thio-dGTP opposite the first two rC_1_rC_2_ positions, but primarily terminates synthesis after extension to rA_3_ (three nucleotides added) prior to translocation, despite the availability of natural dNTPs to add another repeat (Fig. 4c). Telomerase can also add a natural nucleotide (dTTP opposite rA_3_) after 6-thio-dGTP insertion, confirming 6-thio-dGTP is not a chain terminator. However, termination prior to completing synthesis of the 6-nt repeat indicates that 6-thio-dGTP not only disrupts repeat processivity, but also nucleotide processivity. Our data suggest 6-thio-dGTP and 2-OH-dATP addition interfere with telomerase processivity, and compromise translocation and further telomere extension.

### POT1–TPP1 fail to rescue telomerase processivity inhibition

**Fig. 5:**
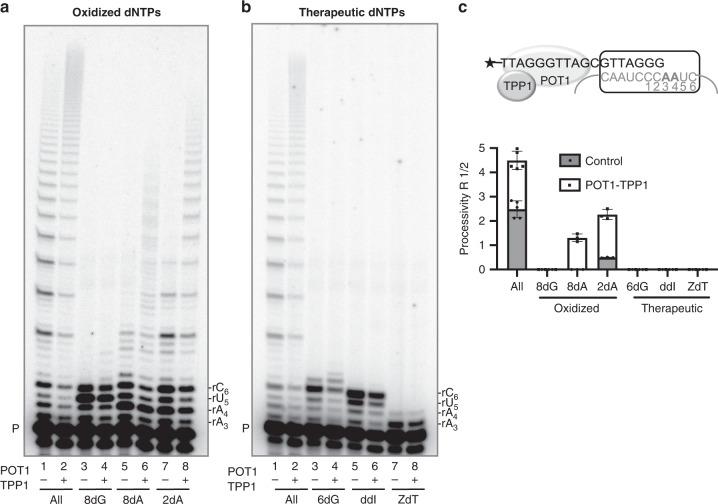
POT1–TPP1 fail to overcome inhibitory dNTPs. Direct telomerase assays were conducted in the absence or presence of 500 nM POT1 and 500 nM TPP1, as indicated, and 5 nM ^32^P-end labeled primer (TTAGGGTTAGCGTTAGGG) designed to position POT1 at the 10 nt primer 5′ end. Reactions contained cellular-concentration dNTPs (lanes 1 and 2), except when the natural dNTP was substituted with the oxidized dNTP **a** or therapeutic dNTP **b** analog (lanes 3–8), as indicated. 8dG (8-oxo-dGTP); 8dA (8-oxo-dATP); 2dA (2-OH-dATP); 6dG (6-thio-dGTP); ddI (ddITP); ZdT (AZT-TP). Letters on the right indicate template base; P indicates unextended 18-mer primer. **c** Processivity was calculated on the basis of total products normalized to loading control. Images are representative of, and data are shown as mean ± s.d. from 3 to 5 independent experiments. Source data are provided as a Source Data file.

Next we tested whether the telomerase processivity factor POT1–TPP1 modulates modified dNTP inhibition of telomerase. POT1 binds to the telomeric ssDNA overhang, requiring the minimum sequence of 5′-TTAGGGTTAG-3′^38^. Inclusion of POT1-binding partner TPP1, increases the binding affinity for telomeric DNA ten-fold compared to POT1 alone^39^. POT1–TPP1 recruits telomerase to telomeres in vivo^39^, and greatly increases telomerase repeat processivity^33^. Therefore, we reasoned that POT1–TPP1 may overcome inhibition by the modified dNTPs and enhance telomerase processivity. We conducted telomerase extension reactions with radiolabeled primer in the presence of POT1–TPP1, and used a primer with a single mutation (TTAGGGTTAGCGTTAGGG; underlined G to C mutation)^40^ to ensure POT1–TPP1 is positioned at the 5′ portion of the primer (Fig. 5). This provides a homogenous substrate for telomerase extension.

For these reactions, we replaced the natural dNTP with the modified dNTP analog, while the other dNTPs remained at cellular relevant concentrations. As a positive control, we show the addition of POT1–TPP1 to telomerase reactions containing all natural dNTPs significantly increased repeat processivity (*R*_1/2_) from 2.5 to 4.5, as indicated by the appearance of longer products (Fig. 5a lanes 1 and 2, and 5c). *R*_1/2_ represents the number of repeats added before half of the DNA substrates dissociate from telomerase, meaning nearly half of the bound primers were elongated by roughly five repeats in the presence of POT1–TPP1. When dGTP was replaced with 8-oxo-dGTP, addition of POT1–TPP1 was unable to overcome the chain termination (Fig. 5a lanes 3 and 4). In contrast, for reactions in which 2-OH-dATP replaced dATP, POT1–TPP1 enhanced repeat processivity 5-fold from 0.5 to 2.3 (Fig. 5a lanes 7 and 8, and 5c). This suggests that 2-OH-dATP incorporation may reduce processivity by disrupting telomerase interaction with the primer, which may be partly compensated for by POT1–TPP1. For reactions in which dATP was replaced with 8-oxo-dATP, we observed some POT1–TPP1 stimulation, although elongation was still greatly reduced compared to control reactions (Fig. 5a lanes 5 and 6). However, we suspect this synthesis resulted from misincorporation of dGTP or dTTP opposite rU_5_ in the absence of dATP (Fig. 3c lanes 2 and 3), since 8-oxo-dATP is a poor telomerase substrate. As expected, POT1–TPP1 failed to stimulate telomerase processivity in reactions containing ddITP and AZT-TP, since insertion of ddITP opposite rC_6_ and AZT-TP opposite rA_3_ halted further synthesis (Fig. 5b lanes 5–8). However, POT1-TPP1 also failed to stimulate telomerase when dGTP was replaced with 6-thio-dGTP (Fig. 5b lanes 3 and 4), indicating this analog is also a strong telomerase inhibitor. Overall, these data indicate that POT1–TPP1 cannot fully restore telomerase extension in the presence of inhibitory dNTPs, although these proteins can partly stimulate processivity in reactions containing 2-OH-dATP.

### Poor selectivity against 6-thio-dGTP ddITP and 2-OH-dATP

In order to better understand the ability of modified dNTPs to compete with natural dNTPs for telomerase utilization, we added modified dNTPs to reactions containing all four natural dNTPs. We conducted reactions with cellular relevant concentrations of natural dNTPs and titrated the modified dNTP from 0 to 10,000 μM to calculate the half-maximal inhibitory concentration (IC_50_) based on telomerase repeat processivity (Supplementary Figs. 8 and 9). The calculated IC_50_ determines the concentration required to reduce repeat processivity in the bulk reactions by half. However, it is important to note that bulk experiments cannot distinguish effects on individual telomeres, since extended telomeres mask unextended telomeres. Incorporation of a chain terminator during any step will terminate extension of the affected telomere, and just five critically short telomeres are sufficient to trigger senescence^41^. Therefore, repeat processivity IC_50_ values are not necessarily an indicator of the inhibitory potency, but provide information on selectivity for modified dNTPs compared to natural dNTPs. The oxidized dNTPs 8-oxo-dATP and 8-oxo-dGTP had similar IC_50_ values as the genuine chain terminator AZT-TP ranging from 402 to 1690 μM (Table 1). However, the therapeutic dNTPs 6-thio-dGTP and ddITP, and oxidized 2-OH-dATP, displayed the lowest IC_50_ values of 5, 61, and 103 μM, respectively. Our data suggest these modified dNTPs, especially 6-thio-dGTP, can effectively compete with the natural dNTPs for binding in the telomerase active site. Given the remarkably low IC_50_ for 6-thio-dGTP, we examined the catalytic efficiency of 6-thio-dGTP incorporation versus dGTP using the *Tribolium castaneum* (*tc*TERT) model of the telomerase catalytic core^42^. The ability to purify sufficient quantities of *tc*TERT enables characterization of the catalytic nucleotide addition by pre-steady-state single turnover kinetics using a defined DNA–RNA primer–template substrate^43^. The catalytic efficiency of incorporating a single dNTP is measured by dividing the observed nucleotide incorporation rate constant, *k*_pol_, by the equilibrium dissociation constant for dNTP binding to the *tc*TERT–primer–template complex (*K*_d_)^44^. The catalytic efficiency of inserting dGTP is only 2-fold higher at 0.034 μM^−1^ s^−1^ compared to 6-thio-dGTP at 0.056 μM^−1^ s^−1^, indicating poor selectivity against this dNTP analog (Table 2). Collectively, these results indicate that modified dNTPs vary in the ability to compete with the natural dNTPs for utilization by telomerase.

**Table 1:** Telomerase processivity IC_50_ values.

Modified dNTP	IC_50_ μM ± SD
8-oxo-dATP	946 ± 49
2-OH-dATP	103 ± 66
8-oxo-dGTP	1690 ± 172
ddITP	61 ± 23
AZT-TP	402 ± 169
6-thio-dGTP	5 ± 2

**Table 2:** Single-turnover kinetic values for tcTERT single nucleotide insertion.

Incoming nucleotide	*k*_pol_ (s^−1^)	*K*_d_ (μM)	Catalytic efficiency (*k*_pol_/*K*_d_) (μM^−1^ s^−1^)	Fold change
dGTP^a^	1.1 ± 0.03	18.1 ± 3.78	0.058 ± 0.012	1.000
6-thio-dGTP	3.1 ± 0.23	92.4 ± 12.94	0.034 ± 0.005	0.579

^a^Values from ref. ^43^.

## Discussion

Treatments with NRTIs and thiopurines, or the failure to remove oxidized dNTPs, drive telomere shortening in human cells^12,13,21,25,28^. Here, we report two distinct mechanisms by which oxidized and therapeutic dNTPs inhibit telomerase-mediated telomere elongation, either by chain termination or by disrupting translocation and subsequent repeat addition. By using end labeled primers, we provide direct evidence that telomerase can add the HIV-1 RT inhibitors ddITP and AZT-TP to a growing telomere chain, and that incorporation halts further elongation. Unlike the NRTIs and 8-oxo-dGTP, we found that telomerase can continue elongation after inserting 2-OH-dATP and 6-thio-dGTP, but that addition of these modified dNTPs strongly inhibits repeat processivity and nucleotide processivity, leading to truncated products. We propose a mechanism by which insertion of these processivity inhibitors disrupt stable formation of the RNA:DNA hybrid required for successful translocation and continued repeat addition.

Defining the mechanism of telomerase inhibition requires analysis of whether telomerase can catalyze addition of the modified dNTP to the telomeric end. Reactions with a single dNTP type also inform about selectivity for inserting a correct dNTP versus an incorrect or modified dNTP during DNA synthesis^44^. Accuracy depends on discrimination against base pairs that cannot adopt Watson–Crick like geometry. Control reactions with natural dNTPs at different template positions revealed features of telomerase accuracy. We found that telomerase incorporation of dGTP is more error prone than the other natural dNTPs, as suggested by dGTP misincorporation at low 5 μM. This propensity for telomerase to misinsert dGTP can outcompete the radioactive dTTP used to label the products in Fig. 2a, explaining the loss of radiolabeled products at high dGTP. Some dTTP misincorporation opposite template rU even at low 5 μM, likely explains the one repeat addition observed when dATP is absent in Fig. 2b. For reactions containing only dGTP, the poly-d(G) ladders appeared at early time points and low percent primer extension (<10%) suggesting they resulted from processive nucleotide addition. Previous reports proposed telomerase synthesis of poly-d(G) ladders is due to DNA hairpin-induced slippage of the product 3′ end relative to the template rC tract^6,45^. Primer slippage may contribute to dGTP addition opposite non-rC residues, although poly-d(G) laddering is not observed at low (5 μM) dGTP, but there is misinsertion opposite rA. High dGTP concentrations can overcome the template-embedded pause site that slows addition of the first dGTP for GGTTAG repeat synthesis^46^. Telomerase has a lower *K*_m_ for dGTP incorporation than other dNTPs, which suggests that telomerase has increased dGTP-binding affinity^37,46–48^, and may explain the higher propensity for dGTP misinsertion and poly-d(G) synthesis. The lower physiological concentration of dGTP (5 μM), relative to the other dNTPs^32^, likely minimizes dGTP misincorporation and laddering in vivo. Furthermore, accessory proteins may assist telomerase fidelity.

The oxidative stress-induced telomere shortening observed upon MTH1 inhibition has been attributed to telomerase 8-oxodGTP insertion which prevents further elongation^12,14^. Our biochemical results indicate that oxidized dATPs can also contribute to telomerase inhibition in MTH1-depleted cells. Although 8-oxo-dATP is a very poor telomerase substrate, it inhibits processivity. Polymerase studies with 8-oxo-dATP are lacking, but mammalian DNA polymerases insert dTTP or dGTP opposite a template 8-oxodA and accommodate the base pairs through base tautomerization and interactions with active site residues^49^. Telomerase may lack contacts required to stabilize an 8-oxodA base pair for catalysis, or 8-oxo-dATP may occupy the active site in a non-productive manner blocking natural dNTP access, similar to telomerase inhibitor 5-MeCITP^50^. In contrast, a 2-OH-dATP:rU base pair is well tolerated in the telomerase active site since 2-OH-dATP insertion opposite rU is only 2-fold less efficient than dATP. 2-OH-dATP can pair with thymine or uracil either in an enol tautomeric form with Watson–Crick hydrogen bonding, or in a keto form through wobble base pairing^51,52^. Similarly, HIV-1 and avian myeoblastosis virus RTs preferentially insert 2-OH-dATP opposite dT or rU more efficiently than replicative polymerases^53,54^. Translesion DNA polymerases harbor a larger binding pocket to accommodate lesions, however, not all can incorporate 2-OH-dATP. Pol η incorporates 2-OH-dATP opposite all template bases except dA, whereas Pol ι cannot^55^. Our data indicate that telomerase insertion of 2-OH-dATP more closely resembles other RTs compared to replicative and TLS polymerases, consistent with a similar binding pocket.

Similar to 2-OH-dATP, we found telomerase readily adds 6-thio-dGTP to the telomeric end and exhibits very poor discrimination against 6-thio-dGTP. First, dGTP and 6-thio-dGTP yielded similar percent extension with all primers tested here. Second, although *tc*TERT has lower affinity for 6-thio-dGTP, as indicated by a higher *K*_d_ than for dGTP, the polymerization rate was faster, yielding similar catalytic efficiencies. Third, 6-thio-dGTP has a very low IC_50_ for telomerase repeat processivity inhibition. This is not surprising given that several human polymerases can insert 6-thio-dGTP with similar efficiencies as dGTP, and structures of Pol β inserting 6-thio-dGTP opposite C, versus dGTP, are nearly identical^56,57^. However, while 6-thio-dGTP can support DNA synthesis, it reduces primer elongation by DNA polymerases compared to dGTP^56^ and strongly inhibits telomerase processivity. While the sulfur substitution has a minor effect on catalysis, it has a dramatic effect on telomere elongation. Our results provide an explanation for why 6-thio-dG promotes telomere shortening in telomerase-positive cells, but not in telomerase-negative cells^25^.

We demonstrate that 2-OH-dATP and 6-thio-dGTP inhibit telomerase by disrupting the translocation step. While insertion of either modified dNTP did not block extension to the next template base, limited synthesis occurred after the first translocation for another repeat addition. Reactions with 6-thio-dGTP terminated immediately prior to, or right after, translocation. Translocation involves DNA:RNA duplex separation and repositioning of the DNA product to reveal the RNA template. Previous evidence suggests the DNA:RNA duplex separates and realigns outside the active site, then the repositioned hybrid binds in the active site^48,58^. Continuous strand separation is thought to maintain the hybrid at a consistent 5 bp length, which is the optimal size for active site accomodation^48^. Protein interactions stabilize this very short hybrid, and telomerase affinity for the hybrid correlates with increased repeat processivity^48^. Therefore, perturbations to the 5 bp hybrid, either via reduced melting temperature or protein interactions, could impact translocation. While substitution of a 6-thio-dG for dG moderately decreases DNA duplex thermal stability, the opening rate of a 6-thio-dG:dC base pair is 17-fold faster than a dG:dC bp^59^. Structures indicate that the hydrogen bond is longer with the thio group and dC, compared to with the carbonyl group, in the Pol β active site^57^. Less is known about how 2-OH-dA impacts bp stability, however, alterations in the bp geometry with rU may impact the telomerase affinity for the RNA:DNA hybrid. Therefore, we proposed minor perturbations could significantly destabilize the already very short, thermally unstable 5 bp RNA:DNA, and thereby disrupt translocation.

POT1–TPP1 results provide further evidence that 2-OH-dATP and 6-thio-dGTP disrupt translocation. POT1–TPP1 enhance telomerase repeat processivity by decreasing the primer dissociation rate and increasing translocation efficiency^40^. POT1–TPP1’s ability to partially restore processivity in the presence of 2-OH-dATP, suggests that 2-OH-dA increases the rate of primer dissociation which is rescued partly by POT1–TPP1 increasing primer binding. However, POT1–TPP1 did not increase processivity in the presence of 6-thio-dGTP, suggesting that 6-thio-dG may be more destabilizing than 2-OH-dA. When 6-thio-dGTP replaces dGTP, each telomeric repeat contains three 6-thio-dGs, whereas when 2-OH-dATP replaces dATP, each repeat contains one 2-OH-dA. Insertion of three modified dNTPs likely disrupts the DNA:RNA to such an extent that POT1–TPP1 enhancement of telomerase primer binding cannot compensate.

Since most tumors rely on telomerase to enable cellular immortality, one strategy to halt proliferation is to target telomerase with inhibitory nucleosides to prevent telomerase restoration of telomeres after each replication. NRTIs exploit the requirement of DNA polymerases and RT for a 3′OH on the ribose to catalyze addition of the incoming dNTP to the growing chain. While AZT-TP was shown to reduce telomerase products in vitro and cause telomere shortening in cells^20,60,61^, our studies provide the first direct evidence that NRTIs are telomerase chain terminators by showing that telomerase catalyzes addition of AZT-TP and ddITP to telomeric DNA. These results have important health implications since AZT has been pursued for anticancer therapy as a telomerase inhibitor^60^. Chronic AZT treatment in mice reduced tumor growth and promoted senescence and apoptosis in mammary carcinoma cells^62^. NRTI inhibition of telomerase has been proposed to promote cellular senescence and features of premature aging in HIV patients undergoing long-term treatments^63^. In addition, several recent preclinical studies showed that 6-thio-dG is an effective treatment for melanoma, lung cancer, and glioblastoma in murine models, and increases telomere dysfunction and shortening^64–66^. Here, we uncover the mechanism by which 6-thio-dGTP compromises telomere maintenance via disrupting telomerase translocation. Finally, the therapeutic dNTPs used in our study not only inhibit telomerase, but also inhibit mitochondrial DNA polymerase γ, leading to mitochondrial dysfunction and elevated ROS which damage dNTP pools^26,27^.Therefore, in conjunction with MTH1 inhibitors, our studies suggest therapeutic NRTIs or thiopurines may deliver a one–two punch to telomerase-driven cancers by inhibiting telomerase directly and indirectly through elevated oxidized dNTPs.

In summary, we show that both oxidized and therapeutic dNTPs inhibit telomerase activity by distinct mechanisms upon incorporation of either chain termination or disruption of telomerase translocation and subsequent repeat addition. Thus, our studies have important health implications for potential off target effects on long-term NRTI treatments, and for therapeutic strategies to target telomerase in cancer.

## Methods

### Telomerase preparation

Telomerase was immunopurified as described^12^ with some modification. HEK-293T (ATCC) cells were grown to 90% confluency in Dulbecco’s modified Eagle’s medium (Gibco) supplemented with 10% FBS (Hyclone) and 1% penicillin–streptomycin (Corning) at 37 °C and 5% CO_2_. Cells were transfected with 10 μg of pSUPER-hTR plasmid and 2.5 μg of pVan107 hTERT plasmid diluted in 625 μl of Opti-MEM (Gibco) mixed with 25 μl of Lipofectamine 2000 (Thermo Fisher) diluted in 625 μl of Opti-MEM. Cells expressing hTR and 3×FLAG-tagged human hTERT were harvested 48 h post-transfection, trypsinized, and washed with PBS, and then lysed in CHAPS buffer (10 mM Tris–HCl, 1 mM MgCl_2_, 1 mM EDTA, 0.5% CHAPS, 10% glycerol, 5 mM β-mercaptoethanol, 120 U RNasin Plus (Promega), 1 μg ml^−1^ each of pepstatin, aprotinin, leupeptin, and chymostatin, and 1 mM AEBSF) for 30 min at 4 °C. Cell lysate supernatant was then flash frozen and stored at −80 °C.

80 μL of anti-FLAG M2 bead slurry (Sigma Aldrich) (per T75 flask) was washed three times with 10 volumes of 1× human telomerase buffer (50 mM Tris–HCl, pH 8, 50 mM KCl, 1 mM MgCl_2_, 1 mM spermidine, and 5 mM β-mercaptoethanol) in 30% glycerol and harvested by centrifugation for 1 min at 3500 rpm and 4 °C. The bead slurry was added to the cell lysate and nutated for 4–6 h at 4 °C. The beads were harvested by 1 min centrifugation at 3500 rpm, and washed 3× with 1× human telomerase buffer with 30% glycerol. Telomerase was eluted from the beads with a 2× the bead volume of 250 μg mL^−1^ 3× FLAG® peptide (Sigma Aldrich) in 1× telomerase buffer containing 150 mM KCl. The bead slurry was nutated for 30 min at 4 °C. The eluted telomerase was collected using Mini Bio-Spin® Chromatography columns (Bio-Rad). Samples were flash frozen and stored a −80 °C.

### Dot blot quantification of telomerase concentration

The concentration of telomerase pseudoknot RNA in the eluted telomerase preparation was measured as described^58^. Briefly, a serial dilution of in vitro transcribed pseudoknot region of hTR (Supplementary Fig. 1) was prepared as standards for quantification (0.1, 0.5, 1, 5, 10, 50, 100, 250 fmol μL^−1^). An aliquot of each standard and eluted telomerase (10 μl) was added to 90 μl of formamide buffer (90% formamide, 1× tris–borate EDTA (TBE)). The samples were incubated at 70 °C for 10 min and then placed on ice. Positively charged Hybond H+ membranes and Whatman filter papers (GE Healthcare Life Sciences) pre-incubated with 1× TBE were assembled onto the GE manifold dot blot apparatus and the samples were loaded onto the membrane via vacuum blotting. The membrane was air dried and then UV-crosslinked using a Stratagene Stratalinker 1800 with the Auto-Crosslink program. The membrane was prehybridized at 55 °C in 25 ml of Church buffer (1% BSA, 1 mM EDTA pH 7.5, 500 mM Na_2_HPO_4_ pH 7.2, 7% SDS) for 30 min. A total of 1 × 10^6^ CPM of ^32^P-labeled hTR oligonucleotide probe (Supplementary Table 1) was added to the hybridization buffer and incubated overnight at 55 °C. The membrane was washed 3× with 0.1× SSC, 0.1× SDS buffer. After vacuum sealing, the membrane was exposed to a phosphorimager screen for 1–3 h and imaged using a Typhoon scanner. ImageQuant TL 8.2 was used to quantify the blot intensities for the standard curve.

### ^32^P-end-labeling of DNA primers

50 pmol of PAGE-purified DNA oligonucleotides (IDT) (Supplementary Table 1) were labeled with γ^32^P ATP (Perkin Elmer) using T4 polynucleotide kinase (NEB) in 1× PNK buffer (70 mM Tris–HCl, pH 7.6, 10 mM MgCl_2_, 5 mM DTT) in a 20 μl reaction volume. The reaction was incubated for 1 h at 37 °C followed by heat inactivation at 65 °C for 20 min. G-25 spin columns (GE Healthcare) were used to purify the end-labeled primer.

### Telomerase activity assay with radiolabeled dNTPs

The telomerase assay was as described^12^. Reactions (20 μl) contained 1× human telomerase buffer, 1 μM oligonucleotide substrate, and dNTP mix as indicated in the figure legends. Reactions with cellular dNTP concentrations contained 24 μM dATP, 29 μM dCTP, 37 μM dTTP, 5.2 μM dGTP, and 0.3 μM 3000 Ci per mmol [α-^32^P] dGTP or [α^32^P] dTTP (PerkinElmer) as indicated. Reactions containing the modified dNTPs (Trilink Biotechnologies) substituted for their natural dNTP analog are indicated in the figure legends. The reactions were started by the addition of 3 μl (~35 fmol) of immunopurified telomerase eluent, incubated at 37 °C for 1 h, then terminated with 2 μl of 0.5 mM EDTA and heat inactivated at 65 °C for 20 min. ^32^P-end labeled 18-mer loading control (8 fmol) was added to the terminated reactions before purification with an Illustra Microspin G-25 column (GE Healthcare). An equal volume of loading buffer (94% formamide, 0.1× TBE, 0.1% bromophenol blue, 0.1% xylene cyanol) was added to the reaction eluent from the G-25 spin column. The samples were heat denatured for 10 min at 100 °C and loaded onto a 14% denaturing polyacrylamide gel (7 M urea, 1× TBE) and electrophoresed for 90 min at constant 38 W. Samples were imaged using a Typhoon phosphorimager (GE Healthcare).

### Telomerase activity assay with end-labeled primers

Reactions (20 μl) contained 1× human telomerase buffer, 5 nM of ^32^P-end-labeled primer and dNTPs as indicated in the figure legends. The reactions were started by the addition of 3 μl of immunopurified telomerase eluent, incubated at 37 °C for 1 h, then terminated with 2 μl of 0.5 mM EDTA and heat inactivated at 65 °C for 20 min. An equal volume of loading buffer (94% formamide, 0.1× TBE, 0.1% bromophenol blue, 0.1% xylene cyanol) was added to the reaction eluent. The samples were heat denatured for 10 min at 100 °C and loaded onto a 14% denaturing acrylamide gel (7 M urea, 1× TBE) and electrophoresed for 90 min at constant 38 W. Samples were imaged using a Typhoon phosphorimager (GE Healthcare). Percent primer extension was calculated with ImageQuant TL 8.2 by measuring the intensity of each product band and dividing by the total radioactivity in the lane or total products, as indicated in the figure legends.

### Quantitation

The processivity was calculated as described^40,67^. Repeat processivity from the direct telomerase extension assays was calculated as *R*_1/2_ (equivalent to the half-time for decay in an exponential time course), which represents the median length of DNA product formed, expressed in terms of number of telomere repeats. First, the total volume counts for each product band extend by one or more telomere repeats were obtained using Image Quant TL. The volume counts were then normalized by dividing by the number of radiolabeled guanosines incorporated into the extended products based on the number of repeats added, termed corrected volume (corr vol). The “percent left behind” (%LB) was calculated for each product band by summing the counts for that product band and for every product band below (shorter products), divided by the total counts for the lane, and then multiplied by 100. The natural log of (100−%LB) was calculated and then plotted vs. repeat number for each product length. A linear regression line was fit to the data to determine the slope of the line. The *R*_1/2_ value was calculated by dividing −ln(2) by the slope of each fitted line (*R*_1/2_ = −ln(2)/slope).

### POT1/TPP1 purification

Full-length human POT1 was expressed as a SUMOstar–hexahistine–POT1 fusion protein in baculovirus-infected SF9 cells (Thermo Fisher Scientific), as described^68^. Sf9 insect cells expressing recombinant POT1 were lysed in buffer (25 mM Tris pH 8.0, 500 mM NaCl, 10 mM imidazole) with a protease inhibitor cocktail (Roche Molecular Biochemicals). Subsequent buffers contained protease inhibitors 2 μg ml^−1^ each of aprotinin, leupeptin, chymostatin, and pepstatin, 1 mM AEBSF and 5 mM β-mercaptoethanol. Following sonication, the lysate was centrifuged at 40,000 r.p.m. for 75 min at 4 °C. The supernatant was filtered through a 0.2 micron filter and loaded onto a HisTrap FF column (GE LifeSciences), followed by washing and elution with 20 and 200 mM imidazole, respectively, using an ATKA Pure FPLC (GE Healthcare). Fractions containing POT1 were pooled and incubated with SUMOstar protease (Ulp1 variant, LifeSensors) for one hour with gentle mixing by rotation 20 rpm at room temperature to cleave the histidine tag. POT1 was separated from the protease and cleaved tag by size-exclusion FPLC chromatography. Samples were loaded on a HiLoad 16/600 Superdex 200 column (GE Healthcare) equilibrated with 25 mM Tris pH 8.0, 150 mM NaCl, 5 mM DTT, and protease inhibitors. Eluted fractions containing POT1 were collected and pooled. Purified TPP1-N (amino acids 89–334) protein was obtained from soluble lysates of isopropyl β-d-thiogalactopyranoside-induced BL21(DE3) pLysS cells (Promega) after nickel agarose chromatography, treatment with Ulp1 protease to cleave the Smt3 tag31 and size-exclusion chromatography as described^69^. Expression was induced with 0.8 mM IPTG in cells for about 13 h at 24 °C, and then harvested by centrifugation at 4500 rpm for 20 min. Cell pellets were lysed in buffer (20 mM Tris pH 7.5, 500 mM NaCl, 10 mM imidazole) with a protease inhibitor cocktail (Roche Molecular Biochemicals). Following sonication, the lysate was centrifuged 40,000 rpm for 75 min at 4 °C. The supernatant was filtered through a 0.2 micron filter and loaded onto a HisTrap FF column (GE LifeSciences), followed by washing and elution with 20 and 200 mM imidazole, respectively, using an ATKA Pure FPLC (GE Healthcare). Fractions containing TPP1 were concentrated and exchanged into buffer (25 mM Tris pH 8.0, 150 mM NaCl, 5 mM DTT) using a Centricon-10 device (Amicon). The sample was incubated with SUMO (Ulp1) protease (Invitrogen) overnight at 4 °C with gentle mixing by rotation at 20 rpm to cleave the tag. Samples were then loaded on a HiLoad 16/600 Superdex 200 column (GE Healthcare). Eluted fractions containing TPP1 were collected and pooled. Protein concentration was determined by Bradford Assay (BioRad) and purity was determined by SDS–PAGE and Coomassie staining.

### Expression and purification of *tc*TERT

*tc*TERT was expressed and purified as described with some modifications^42,43^. An Epiphyte3 LEX bioreactor was used to grow *tc*TERT in BL-21(DE3) pLysS cells at 37 °C until they reached an OD_600_ of 0.6–0.8, after which protein expression was induced with 1 mM isopropyl β-d-1-thiogalactopyranoside (IPTG) and the temperature was decreased to 30 °C for 4-5 hours of protein induction. Cells were harvested via centrifugation at 4000 × *g* until lysis. For *tc*TERT purification, buffers containing 0.75 M KCl and 10% glycerol was used for the initial purification step on Ni-NTA columns (GE Healthcare). Samples were further purified via cation exchange on a POROS HS column (Thermo Fisher), using a salt gradient of 0.5 M KCl to 1.5 M KCl. Next, the hexahistidine tag was cleaved with Tobacco etch virus (TEV) protease overnight at 4 °C. The cut tag and TEV protease were separated from the protein with an additional Ni-NTA column chromatography step. The final chromatography step was a Sephacryl S-200 16/60, GE Healthcare column using a buffer containing 50 mM Tris–HCl, pH 7.5, 10% glycerol, 0.8 M KCl, and 1 mM Tris(2-carboxyethyl)phosphine (TCEP). Resultant *tc*TERT was concentrated down to 18 mg ml^−1^ and stored at 4 °C^42^.

### Pre-steady-state kinetics of *tc*TERT inserting 6-thio-dGTP

Pre-steady-state kinetic parameters of *tc*TERT were obtained using established pre-steady-state kinetics protocols for DNA polymerases, also known as single turnover kinetics^70,71^. Briefly, we preincubated 2 μM *tc*TERT with 200 nM annealed DNA:RNA hybrid substrate, with a 6-FAM label on the 5′ end of the DNA component. We then used a KinTek RQF-3 (a rapid quench-flow instrument) to mix equal ratios of the incoming nucleotide triphosphate and 10 mM MgCl_2_ with the existing mix of *tc*TERT and its DNA:RNA hybrid substrate. Reactions were run at 37 °C and quenched at various timepoints with 100 mM EDTA pH 7.5. In each case, the conditions used for each reaction were: 25 mM Tris pH 7.5, 0.05 mg ml^−1^ bovine serum albumin, 1 mM dithiothreitol, 10% glycerol, 200 mM KCl, 1 μM *tc*TERT, 100 nM annealed DNA:RNA hybrid substrate, and varying concentrations of 6-thio-dGTP. After each reaction, the samples were transferred to a DNA gel loading buffer, containing 100 mM EDTA, 80% deionized formamide, 0.25 mg ml^−1^ bromophenol blue, and 0.25 mg ml^−1^ xylene cyanol. These mixes were then incubated at 95 °C for 5 min, and loaded onto a 21% denaturing polyacrylamide gel. These gels were run at 700 V, 60 A, and 30 W at 30 °C in order to separate the reaction product from its substrate.

Gels were scanned and imaged using a GE Typhoon FLA 9500 imager, and the ratios of product to substrate were quantified using ImageJ version 1.52k^72^. Means and standard deviations from at least three independent replicates were calculated, and graphed using KaleidaGraph version 4.5.2. Plots of product formation over time were fit to the exponential equation (1) to determine *k*_obs_ values:

1 $$\left[ {P} \right] = {A}(1 - {\mathrm{e}}^{ - k_{{\mathrm{obs}}}t}).$$

In which [*P*] is the concentration of the product, *A* is the target engagement (amplitude), and *t* is the reaction time. After *k*_obs_ values were determined for multiple nucleotide triphosphate concentrations, the data was replot to compare *k*_obs_ to concentration of nucleotide triphosphate, and fit to Eq. (2):

2 $$k_{{\mathrm{obs}}} = \frac{{k_{{\mathrm{pol}}}[{\mathrm{NTP}}]}}{{K_{\mathrm{d}} + [{\mathrm{NTP}}]}}.$$

with *k*_pol_ representing the theoretical maximum value of *k*_obs_, and [NTP] representing the concentration of the nucleotide of interest.

### Polymerase β dGTP run-on assay

40 nM polymerase β, 400 nM primer–template DNA with a 5′ 6-FAM-labeled PolB Primer 1 annealed to PolB Primer 2 (Supplementary Table 1), were preincubated for 20 min. We then used a multi-channel pipette to mix equal ratios of the incoming nucleotide with 1 mM MgCl_2_ to start the reaction. Reactions were run at 37 °C in a LabDoctor™ heating and quenching was accomplished using a solution of DNA gel-loading buffer. In each reaction the conditions were: 50 mM Tris pH 7.5, 0.1 mg ml^−1^ bovine serum albumin, 1 mM dithiothreitol, 10% glycerol, 100 mM KCl, 20 nM polymerase β, 200 nM primer template DNA, and either 50 or 200 μM dGTP or dTTP. These mixes were then incubated at 95 °C for 5 min, and loaded onto a 21% denaturing polyacrylamide gel. These gels were run at 700 V, 60 A, and 30 W at 30 °C in order to separate the reaction product from its substrate. Gels were scanned and imaged using a GE Typhoon FLA 9500 imager.

### Statistics

Means and standard deviations were calculated using Graph Pad Prism version 8.

### Reporting summary

Further information on research design is available in the Nature Research Reporting Summary linked to this article.

## Supplementary information

Supplementary Information

Reporting Summary

## Data Availability

Supplementary Information including uncropped images are included in the online version of this manuscript. All data supporting the findings of this study are available from the corresponding author upon reasonable request. Source data are provided with this paper.

## Source data

Source Data
