# Supplementary material for: Mechanisms of telomerase inhibition by oxidized and therapeutic dNTPs
Source: Nat Commun. 2020 Oct 20;11:5288. doi: 10.1038/s41467-020-19115-y (PMC7576608; doi:10.1038/s41467-020-19115-y)

Supplementary Information for

# Mechanisms of telomerase inhibition by oxidized and therapeutic dNTPs.

Sanford et al.

*Corresponding author. Email: plo4@pitt.edu

**The PDF file includes:**

**Supplementary Figure 1: Quantification of immunopurified telomerase-protein-RNA**
**Supplementary Figure 2: Telomerase dNTP titrations and processivity calculations**
**Supplementary Figure 3: Telomerase extension with dGTP**
**Supplementary Figure 4: Time course reactions with telomerase or pol β**
**Supplementary Figure 5: Telomerase extension with natural dNTPs**
**Supplementary Figure 6: Telomerase extension with oxidized dNTPs**
**Supplementary Figure 7: Telomerase extension with therapeutic dNTPs**
**Supplementary Figure 8: $IC_{50}$ values for telomerase processivity inhibition**
**Supplementary Figure 9: Representative titration gels for $IC_{50}$ values**

**Supplementary Table 1: Oligonucleotide sequences used in this study**

**Supplementary Figure 10: Uncropped gels from Figure 3**
**Supplementary Figure 11: Uncropped gels from Figure 4**
**Supplementary Figure 12: Uncropped gels from Supplementary Figure 1c**
**Supplementary Figure 13. Uncropped gels from Supplementary Figure 5**
**Supplementary Figure 14. Uncropped gels from Supplementary Figure 6**
**Supplementary Figure 15. Uncropped gels from Supplementary Figure 7**

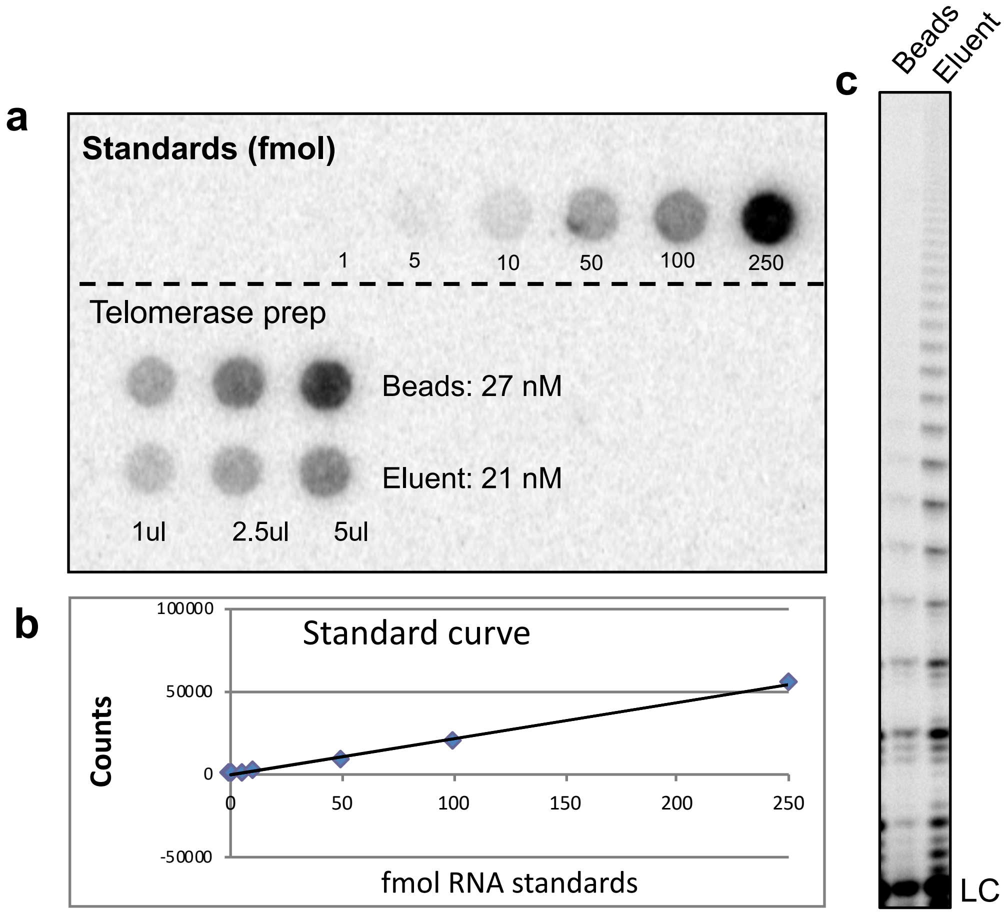


**Supplementary Fig 1. Quantification of immunopurified telomerase-protein-RNA complexes.** (a) Telomerase was over expressed in HEK293T cells and immunopurified (IP) using a FLAG tag on the N-terminus of TERT. The beads or eluate from IP reactions were spotted on a blot and probed with a $^{32}$P labeled probe against hTR to determine the concentrations of RNA that co-purified with TERT. 1, 2.5, and 5 µL aliquots were compared with *in vitro* transcribed hTR standards from 0.5 to 250 fmol. Concentrations are shown. (**b**) Standard curve of hTR standards to determine concentration of telomerase. (**c**) Telomerase primer extension assays. Telomerase activity from beads and eluent preparations were compared using a $(TTAGGG)_3$ primer. LC indicated 18-mer loading control. Images represent dot blot for one telomerase prep. Source data are provided as a Source Data file.

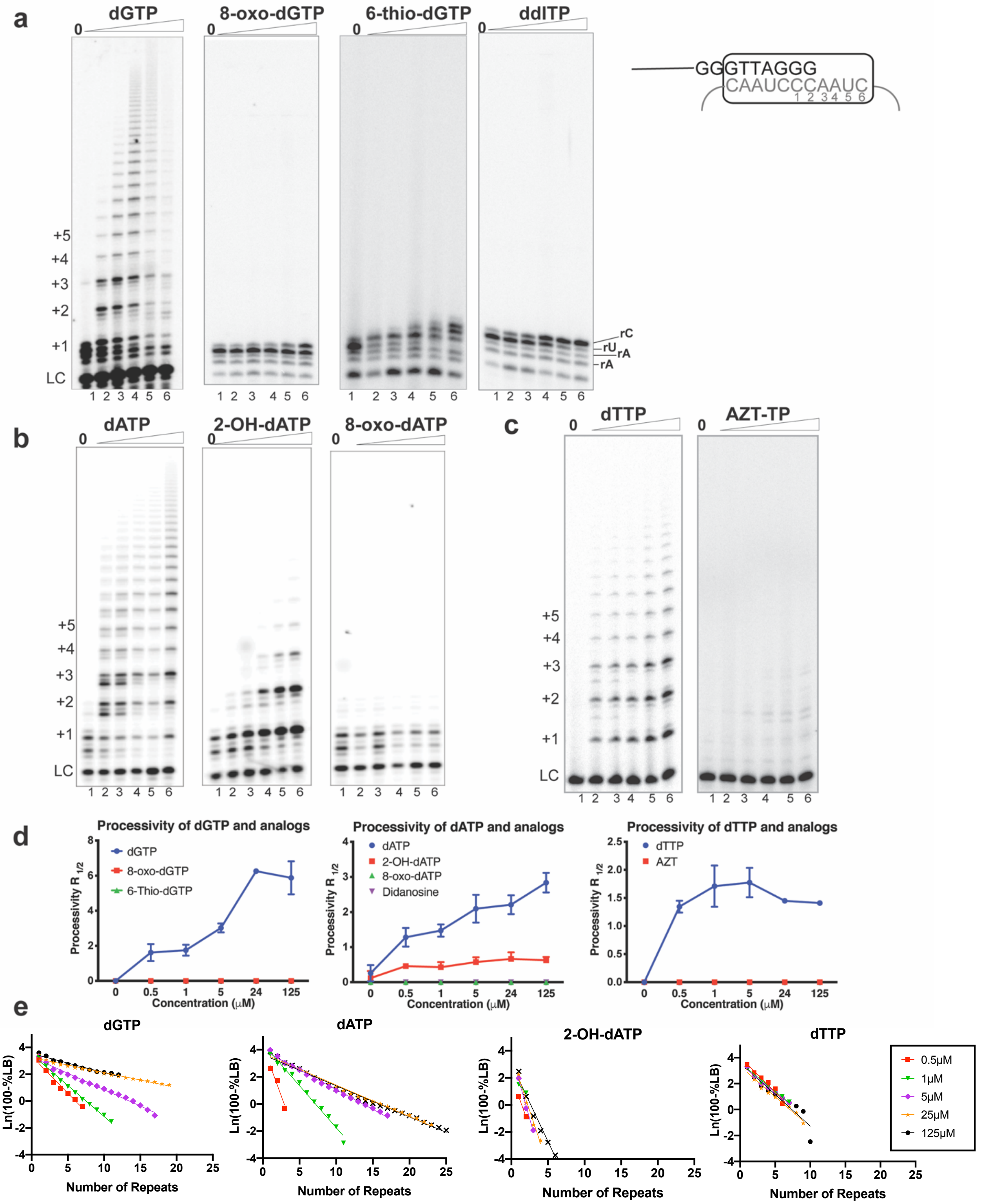


**Supplementary Fig 2.Telomerase dNTP titrations and processivity calculations.** Telomerase reactions were conducted with $(TTAGGG)_3$ primer 1 and cellular-concentration dNTPs except that the indicated natural or modified dNTP was added at increasing concentrations (0, 0.5, 1, 5, 25, and 125 μM) along with either 0.3 μM [α-$^{32}$P]dTTP (**a**) or [α-$^{32}$P] dGTP (**b, c**). The loading control (LC) was a $^{32}$P-end labeled 18-mer. Numbers on the left indicate the number of added repeats. **(d)** Processivity was calculated as $R_{1/2}$ (see Methods). (**e**) The natural log of (100-%LB) was plotted vs repeat number. A straight line was fit to the data and the $R_{1/2}$ was calculated by dividing –ln(2) by the slope of each line. Plots shown from one experiment. Images (**a-c**) and plots (**e**) are representative of, and data are mean ± s. d. (**d**) from, three independent experiments. Source Data are provided as a Source Data file.

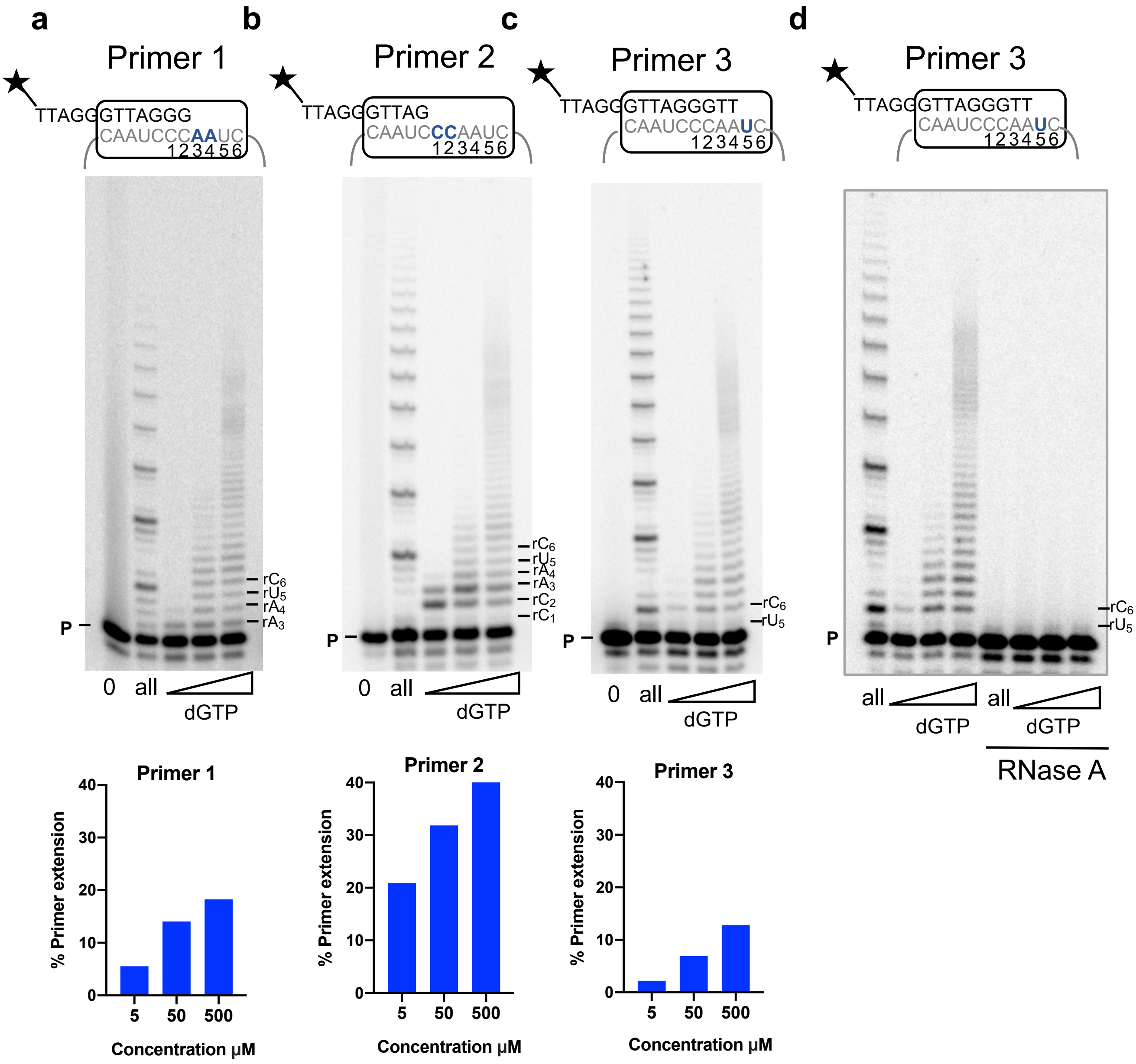


**Supplementary Fig 3.Telomerase extension with dGTP.** Telomerase reactions were conducted with 5 nM $^{32}$P-end labeled primer (**a**) Primer 1 $(TTAGGG)_3$, (**b**) Primer 2 $(GGTTAG)_3$, or (**c** and **d**) Primer 3 $(AGGGTT)_3$. Reactions contained cellular-concentration dNTPs (all) or 5, 50, or 500 µM dGTP. In panel (**d**) reactions contained 3 µg/µl RNase A. Products were separated on denaturing gels. Letters on the right indicate the template base; P indicates unextended 18-mer primer. Graphs represent the percent of total primers extended for the reactions in panels **a** – **c** from one experiment. Panel **d** is a representative image from three independent experiments. Source data are provided as a Source Data file.

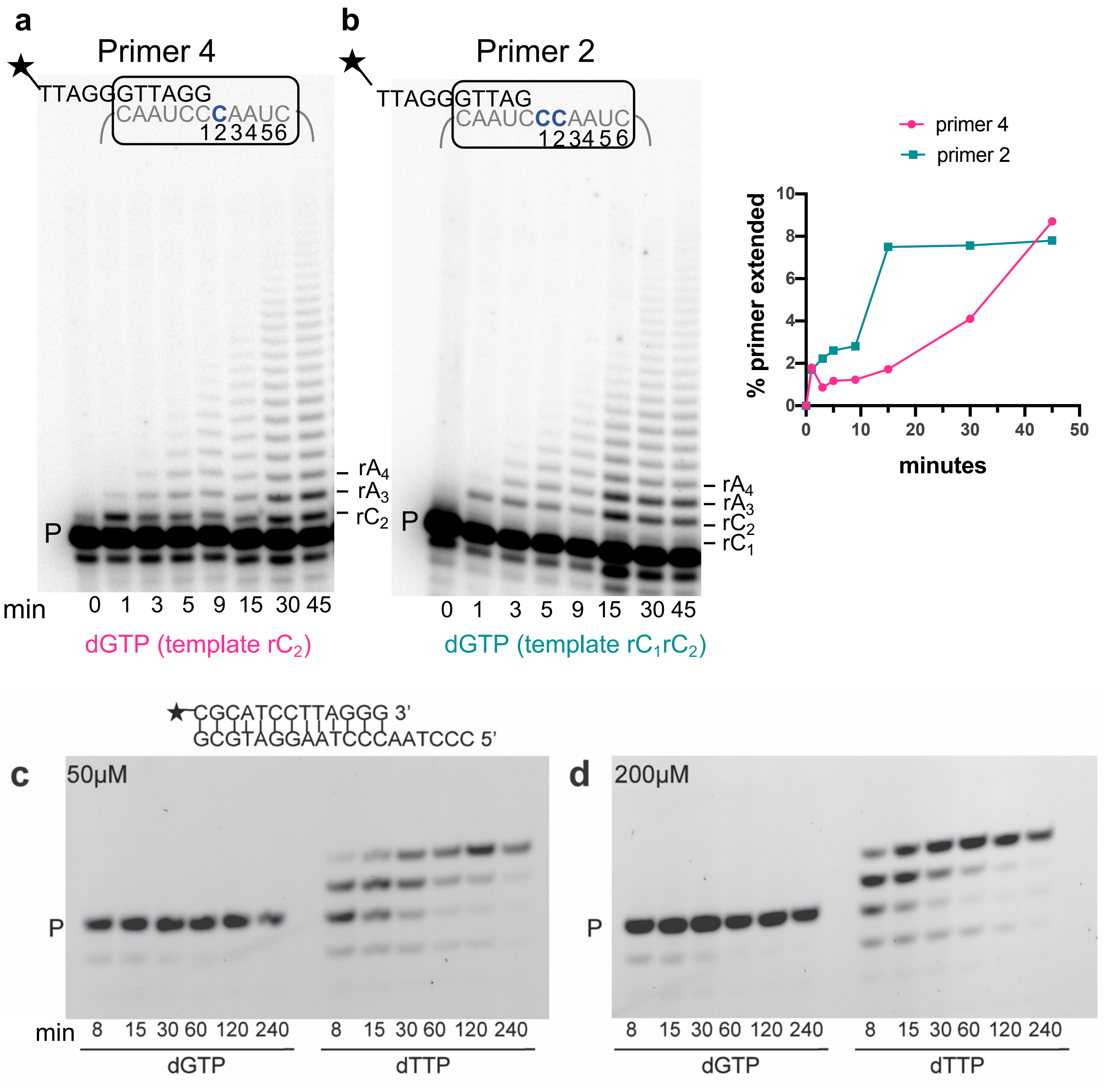


**Supplementary Fig 4. Time course reactions with telomerase or pol β. (a, b)** Telomerase reactions were conducted with 5 nM $^{32}$P-end labeled primer (**a**) Primer 4 $(GTTAGG)_3$ or (**b**) Primer 2 $(GGTTAG)_3$. Reactions contained 50 µM dGTP and were terminated at 0, 1, 3, 5, 9, 15, 30, or 45 minutes as indicated. Products were separated on denaturing gels. Letters on the right indicate the template base; P indicates unextended 18-mer primer. Graphs represent the percent primer extended for the reactions in panels **a** and **b** from one experiment. (**c, d**) Primer extension by human polymerase β. The double stranded 6-FAM labeled primer-template and polymerase β were incubated with either 50 µM dGTP or dTTP (**c**), or 200 µM dGTP or dTTP (**d**) for time ranging from 8-240 minutes as indicated. The products of the reactions were run on denaturing gels and imaged using a Typhoon phosphoimager. P indicates unextended 13-mer primer from three independent experiments. Source data are provided as a Source Data file.

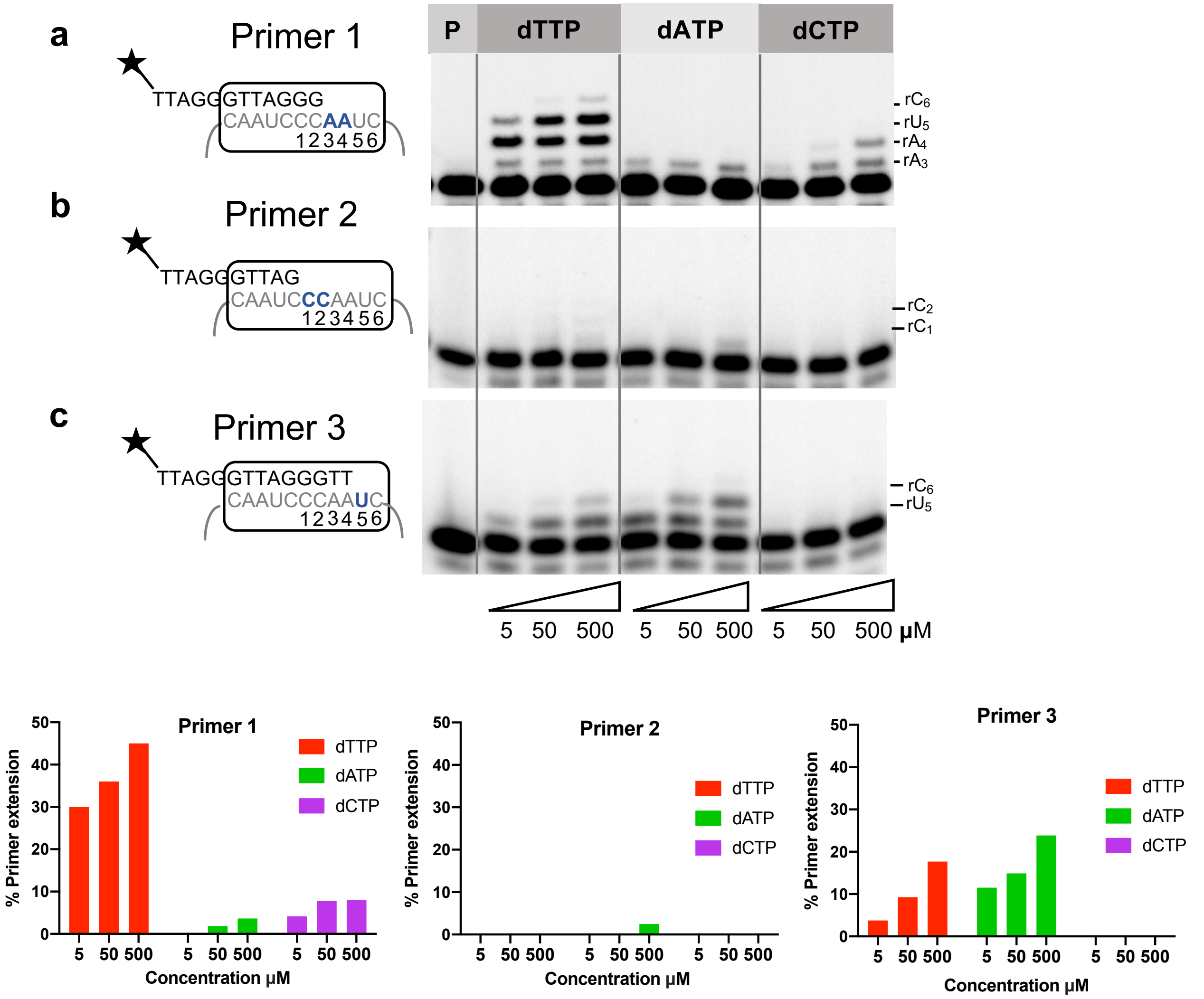


**Supplementary Fig 5. Telomerase extension with natural dNTPs.** Telomerase reactions were conducted with 5 nM $^{32}$P-end labeled primer (**a**) Primer 1 (TTAGGG)$_3$, (the box around dTTP reactions indicates image from a separate gel) (**b**) Primer 2 (GGTTAG)$_3$, or (**c**) Primer 3 (AGGGTT)$_3$. Reactions contained 5, 50, and 500 µM dTTP, dATP, or dCTP as indicated. Products were separated on denaturing gels. Letters on the right indicate the template base; P indicates unextended 18-mer primer. Graphs represent the percent of total primers extended for the reactions in panels **a**, **b**, and **c** from one experiment. Source data are provided as a Source Data file.

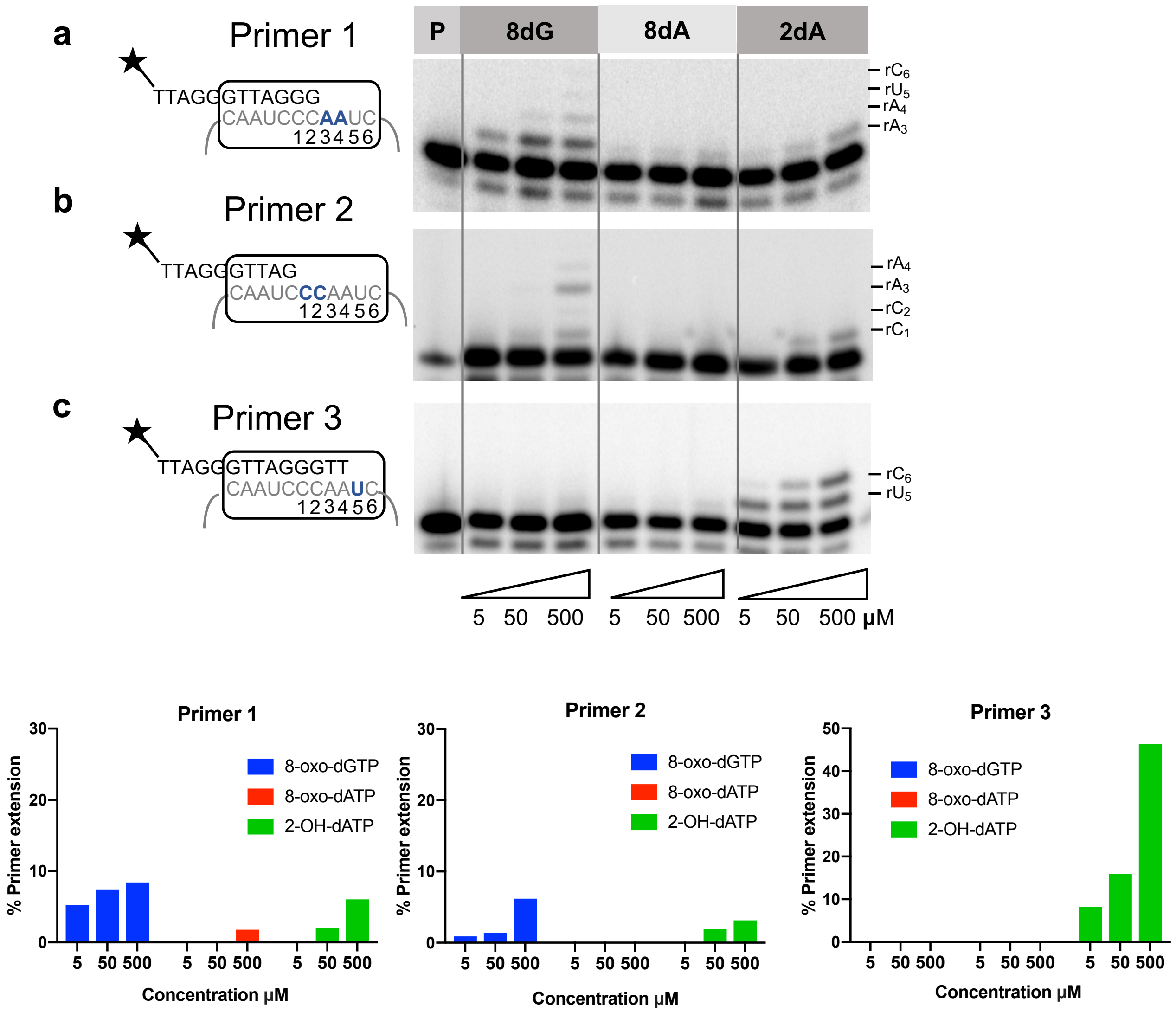


**Supplementary Fig 6. Telomerase extension with oxidized dNTPs.** Telomerase reactions were conducted with 5 nM $^{32}$P-end labeled primer (**a**) Primer 1 $(TTAGGG)_3$, (**b**) Primer 2 $(GGTTAG)_3$, or (**c**) Primer 3 $(AGGGTT)_3$. Reactions contained 5, 50, and 500 µM 8-oxo-dGTP (8dG), 8-oxo-dATP (8dA), or 2-OH-dATP (2dA) as indicated. Products were separated on denaturing gels. Letters on the right indicate the template base; P indicates unextended 18-mer primer. Graphs represent the percent of total primers extended for the reactions in panels **a**, **b**, and **c** from one experiment. Source data are provided as a Source Data file.

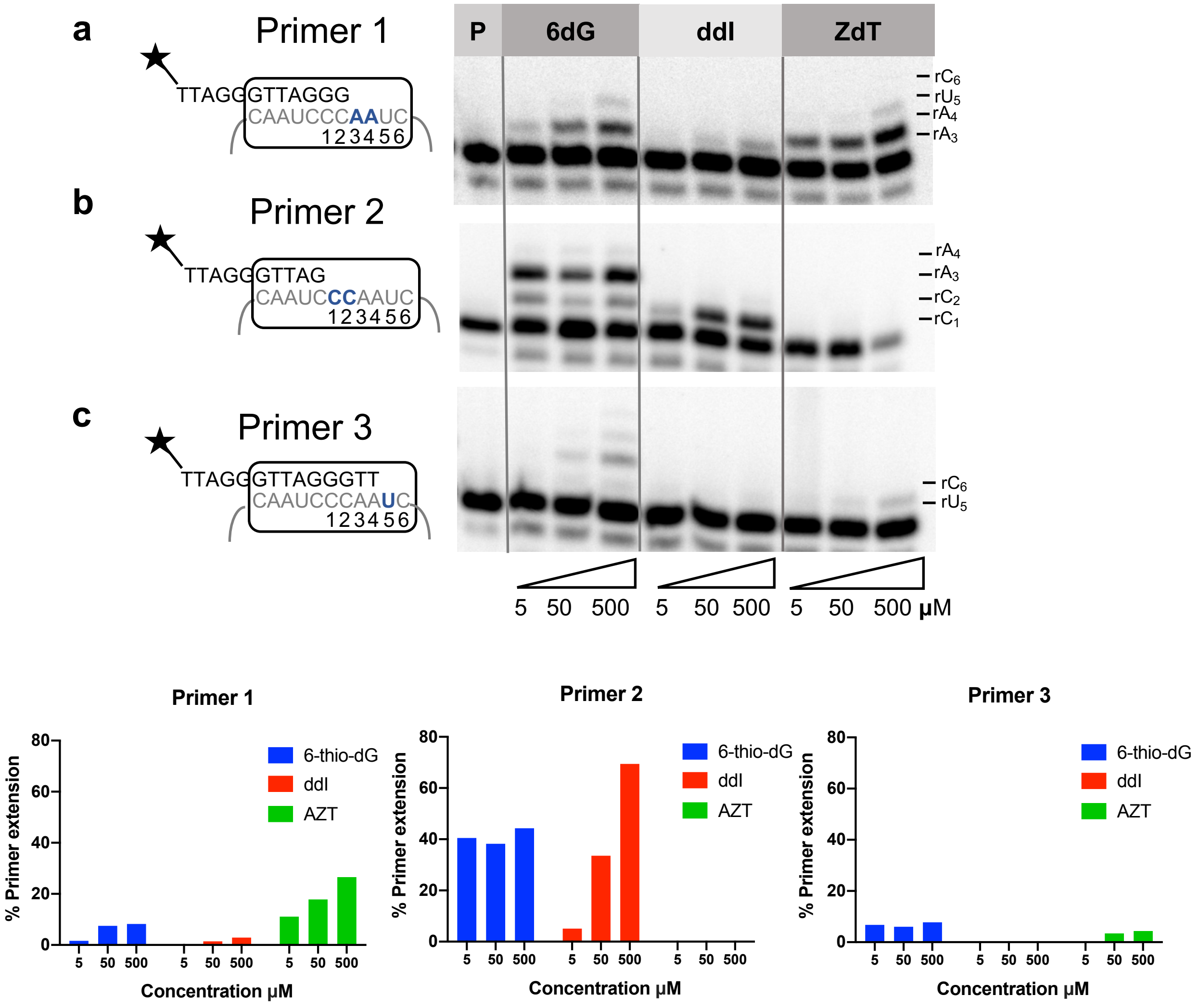


**Supplementary Fig 7. Telomerase extension with therapeutic dNTPs.** Telomerase reactions were conducted with 5 nM $^{32}$P-end labeled primer (**a**) Primer 1 $(TTAGGG)_3$, (**b**) Primer 2 $(GGTTAG)_3$, or (**c**) Primer 3 $(AGGGTT)_3$. Reactions contained 5, 50, and 500uM 6dG (6-thio-dGTP), ddI (ddITP) or ZdT (AZT-TP) as indicated. Products were separated on denaturing gels. Letters on the right indicate the template base; P indicates unextended 18-mer primer. Graphs represent the percent of total primers extended for the reactions in panels **a**, **b**, and **c** from one experiment. Source data are provided as a Source Data file.

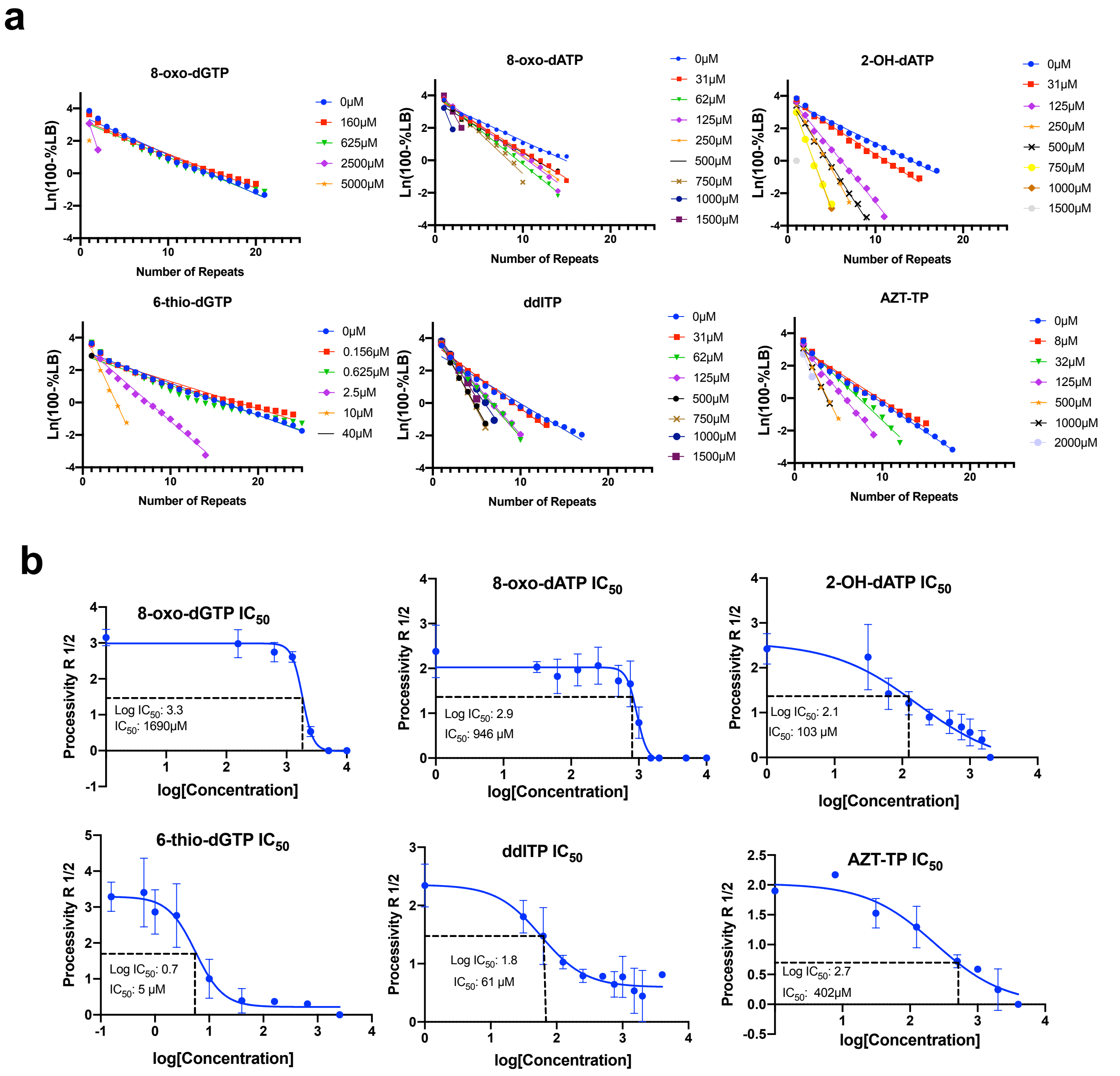


**Supplementary Fig 8. $IC_{50}$ values for telomerase processivity inhibition.** Telomerase reactions were conducted with cellular relevant concentrations of all four natural dNTPs and increasing concentrations of the modified dNTP analog from 0 to 10,000 μM to calculate. (**a**) The natural log of (100-%LB) was plotted vs repeat number. A straight line was fit to the data and the $R_{1/2}$ value was calculated by dividing –ln(2) by the slope of each line. Plots shown from one experiment are representative of 3-4 independent experiments, and from 2 experiments for AZT-TP reactions. (**b**) The half maximal inhibitory concentration ($IC_{50}$) based on telomerase processivity. Means and s.d. are from 3-4 independent experiments, and from 2 experiments for AZT-TP reactions. Source data are provided as a Source Data file.

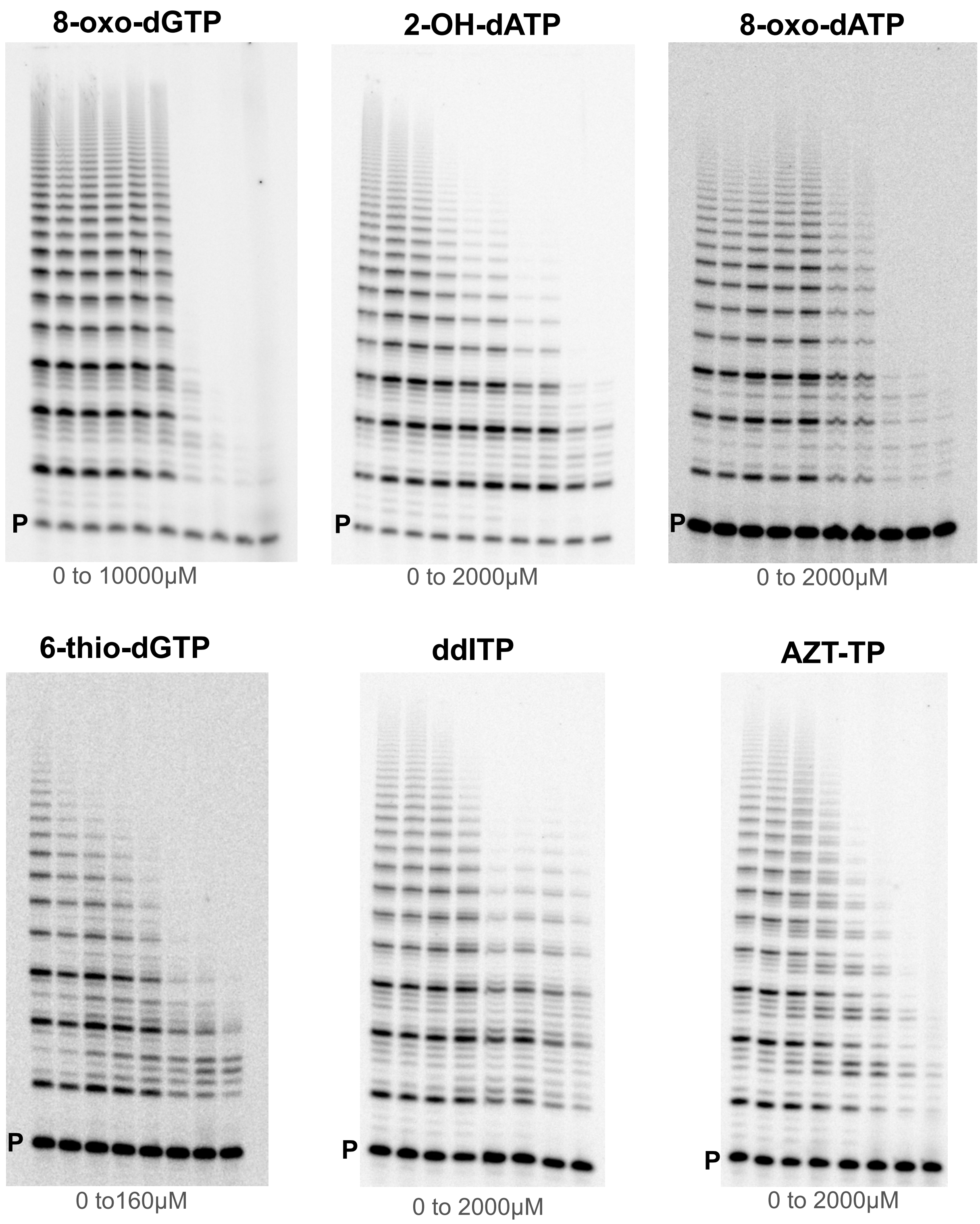


**Supplementary Fig 9. Representative titration gels for $IC_{50}$ values.** Telomerase reactions were conducted with cellular relevant concentrations of all four natural dNTPs and increasing concentrations of the modified dNTP analog from 0 to 10,000 µM. Products were separated on denaturing gels; P indicates unextended 18-mer primer. Images are representative from 3-4 independent experiments, and from 2 experiments for AZT-TP reactions.

# Supplementary Table 1. Oligonucleotide sequences used in this study

| Primer name | Sequence (5' to 3') |
|---|---|
| Primer 1 (template $rA_3rA_4$) | TTAGGGTTAGGGTTAGGG |
| Primer 2 (template $rC_1rC_2$) | GGTTAGGGTTAGGGTTAG |
| Primer 3 (template $rU_5$) | AGGGTTAGGGTTAGGGTT |
| Primer 4 (template $rC_1$) | GTTAGGGTTAGGGTTAGG |
| RNA dot blot probe | CGGTGGAAGGCGGCAGGCCGAGGC |
| hTR PCR Primer forward | TAATACGACTCACTATAGGGCCATTTTTTGTCTAACCC |
| hTR PCR Primer reverse | AACGGGCCAGCAGCTG |
| Primer A5 | TTAGGGTTAGCGTTAGGG |
| PolB Primer 1 | /56-FAM/CGCATCCTTAGGG |
| PolB Primer 2 | CCCTAACCCTAAGGATGCG |
| TcTERT Primer 1 | /56-FAM/CCAGCCAGGTCAG |
| TcTERT Primer 2 | GGUCGGUCCAGUCCAGU |

## Supplementary Figure 10. Uncropped gels from Figure 3

**3a**

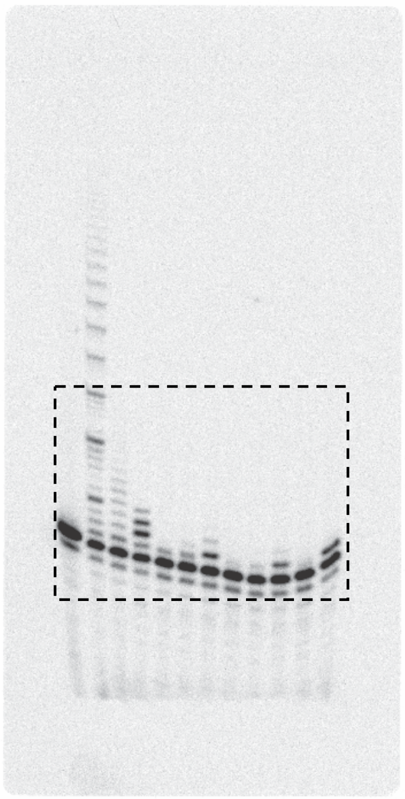

**3b**

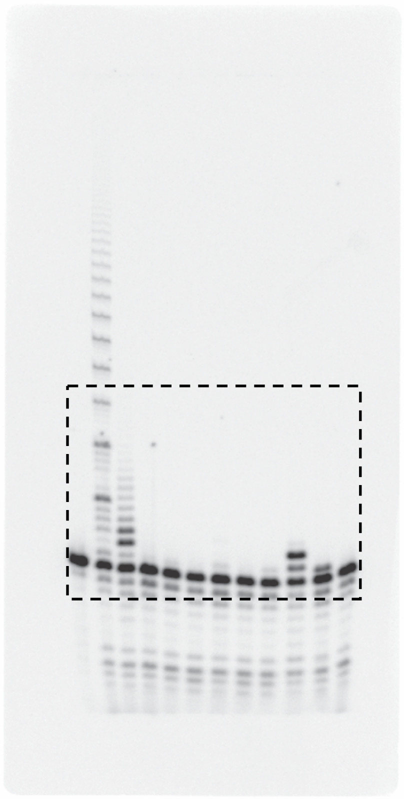

**3c**

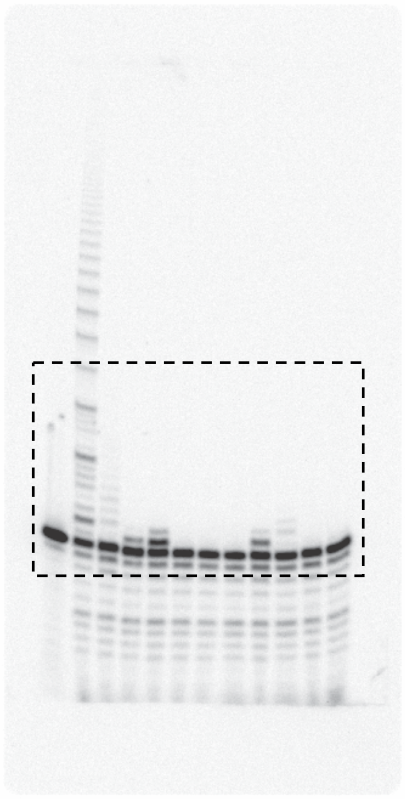

## Supplementary Figure 11. Uncropped gels from Figure 4

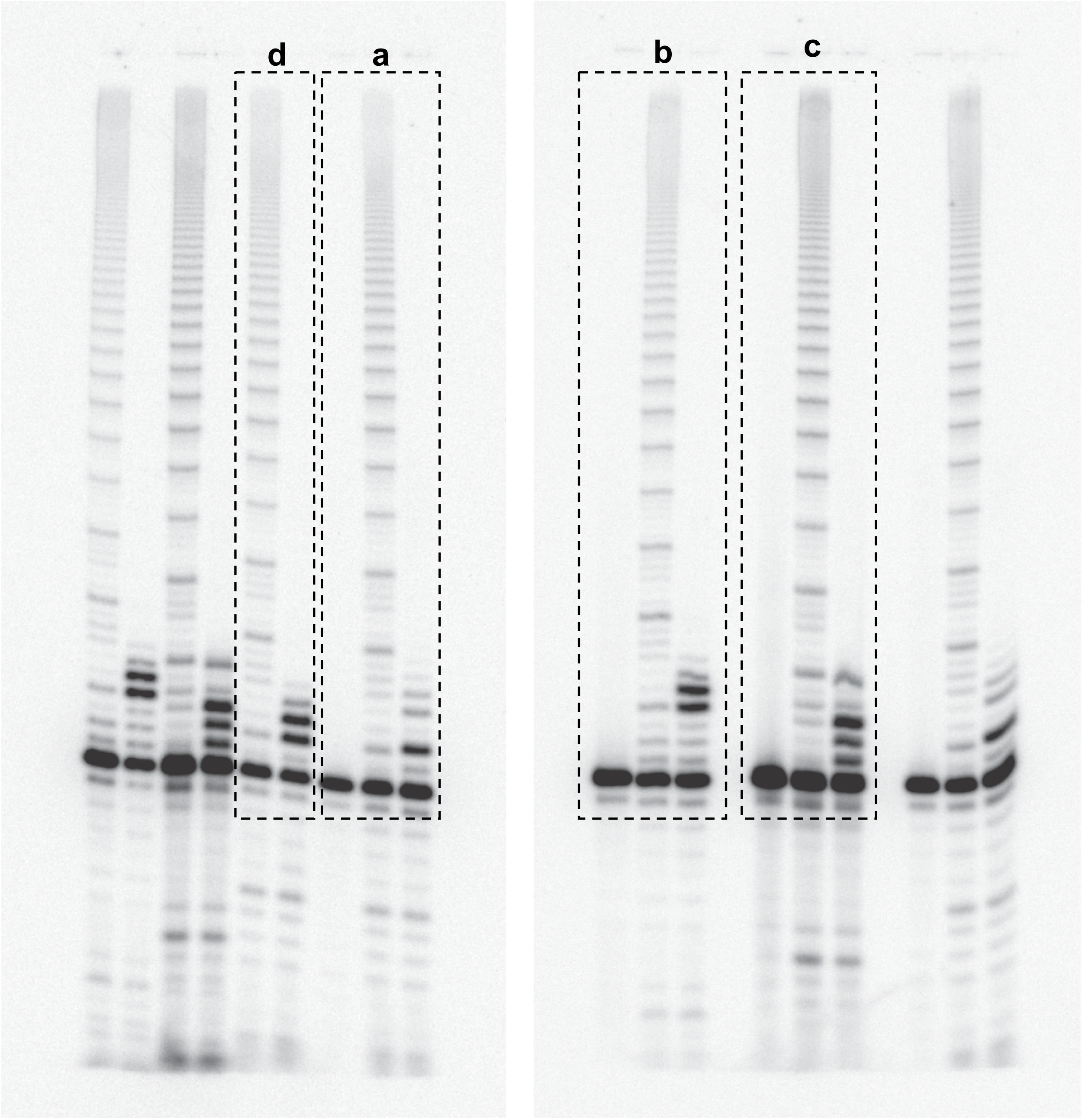

**Supplementary Figure 12. Uncropped gel from Supplementary Figure 1c**

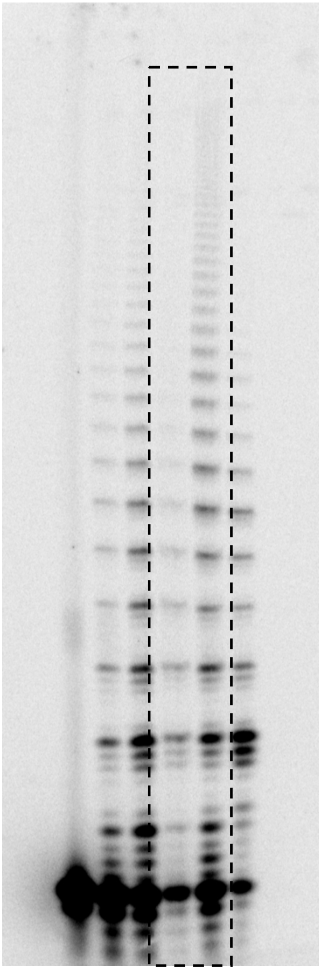

# Supplementary Figure 13. Uncropped gels from Supplementary Figure 5

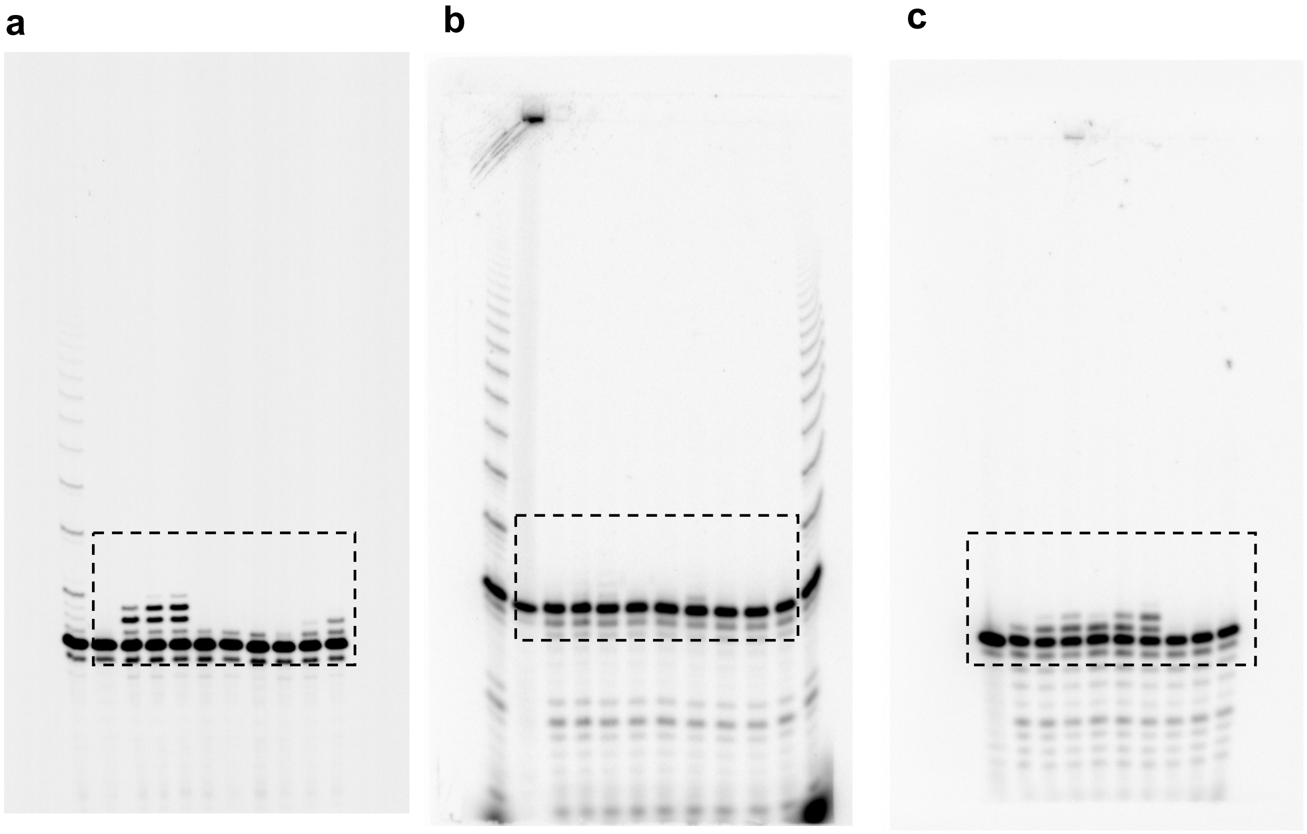

## Supplementary Figure 14. Uncropped gels from Supplementary Figure 6

a

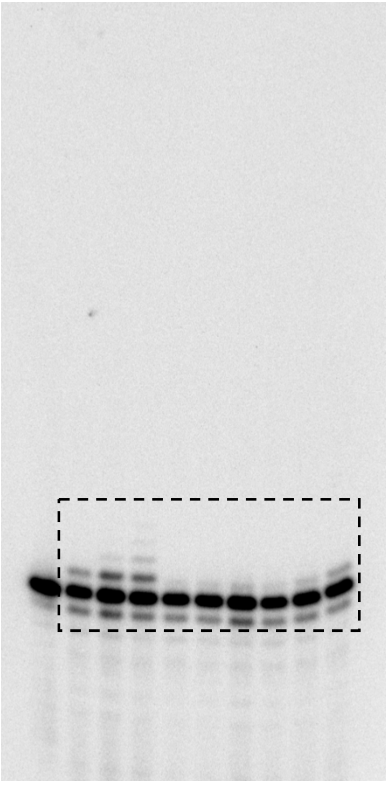

b

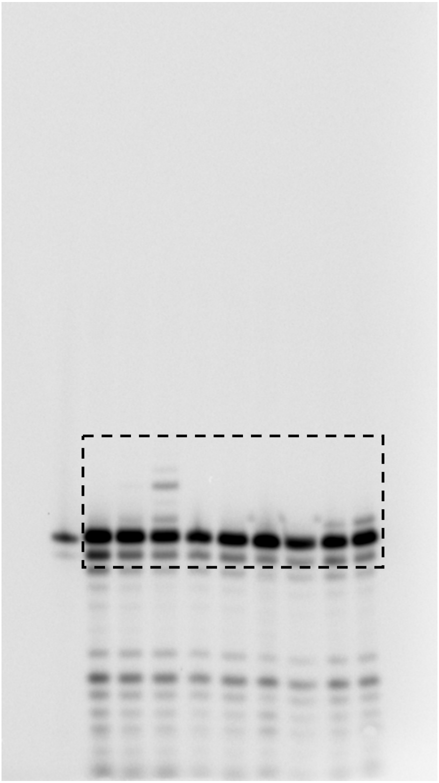

c

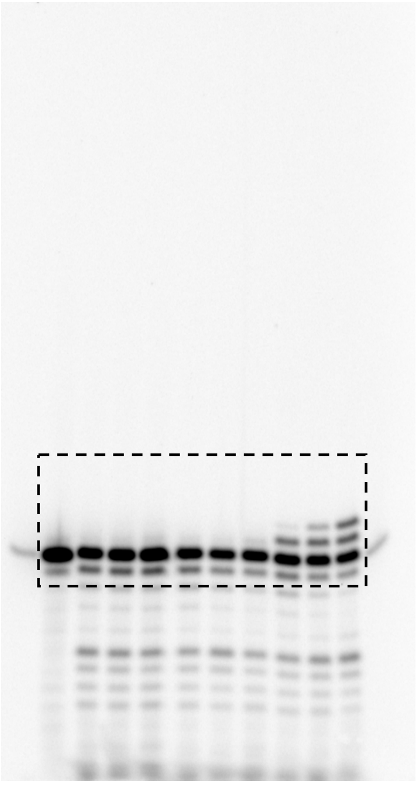

**Supplementary Figure 15. Uncropped gels from Supplementary Figure 7**

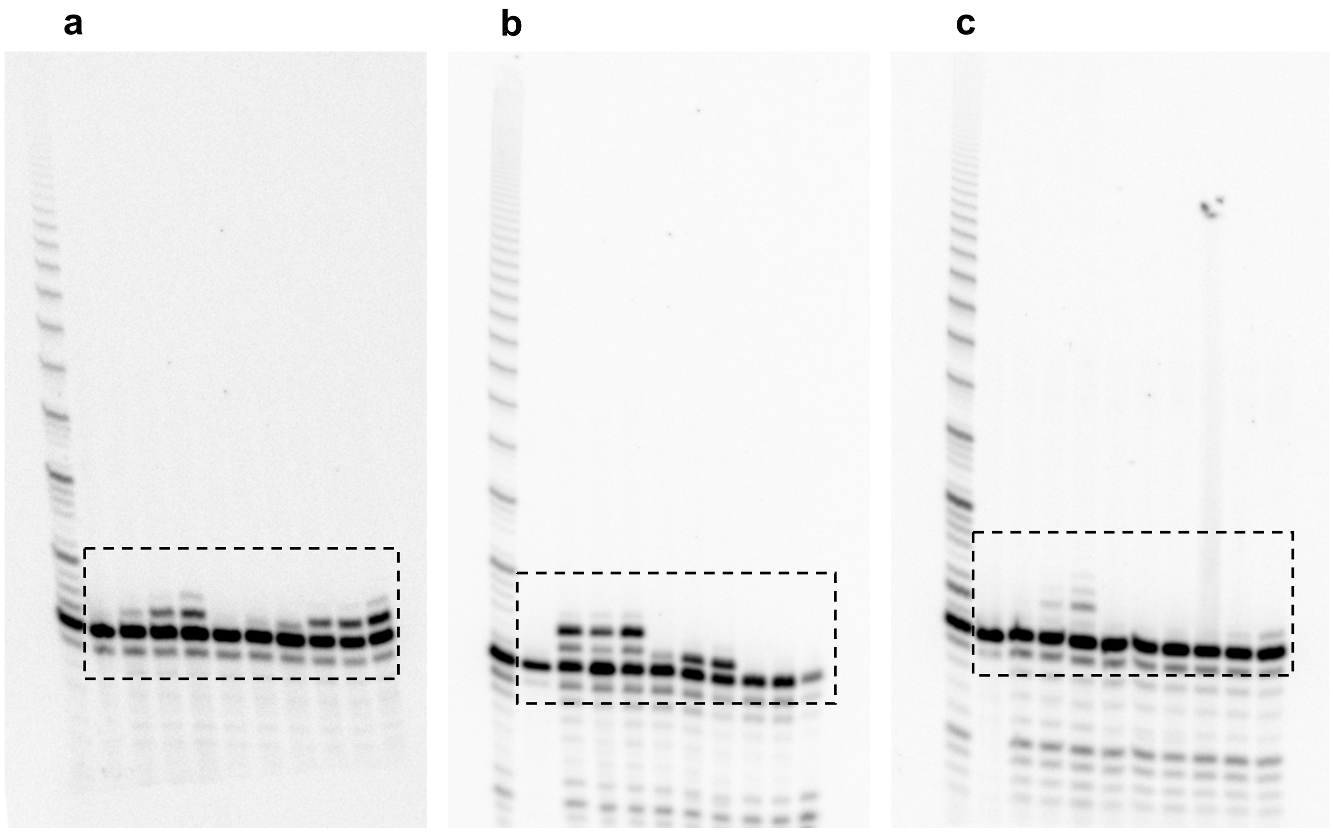

Supplement: Supplementary file 1 — Supplementary Information [file 41467_2020_19115_MOESM1_ESM.pdf]
